# Supplementary material for: Elimination of Unfit Cells Maintains Tissue Health and Prolongs Lifespan
Source: Cell. 2015 Jan 29;160(3):461–76. doi: 10.1016/j.cell.2014.12.017 (PMC4313366; doi:10.1016/j.cell.2014.12.017)
Supplement: Document S1. Article plus Supplemental Information [file mmc1.pdf]

Cell

Article

Elimination of Unfit Cells Maintains Tissue Health and Prolongs Lifespan

Graphical Abstract

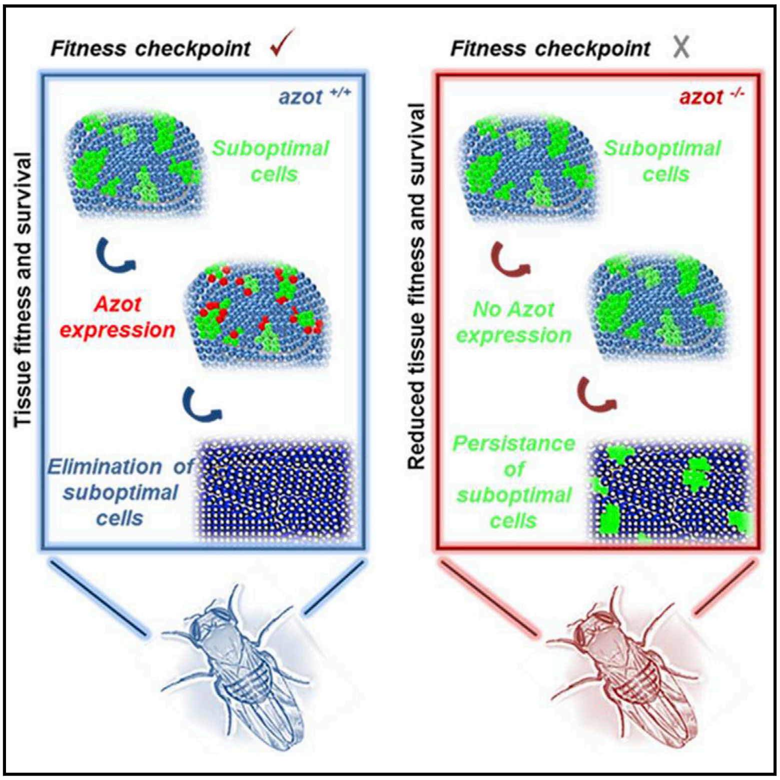

Authors

Marisa M. Merino, Christa Rhiner, ..., Barbara Hauert, Eduardo Moreno

Correspondence

emoreno@izb.unibe.ch

In Brief

Elimination of less fit cells ensured by the expression of a cell-fitness checkpoint, Azot, is important for maintaining tissue health and prolonging lifespan in *Drosophila*.

Highlights

- Fitness-based cell culling maintains tissue health
- Azot ensures the elimination of less fit cells
- Lack of *azot* accelerates tissue degeneration
- Improving the efficiency of cell selection extends lifespan

Merino et al., 2015, Cell *160*, 461–476
January 29, 2015 ©2015 The Authors
http://dx.doi.org/10.1016/j.cell.2014.12.017

CellPress

Cell

Elimination of Unfit Cells Maintains Tissue Health and Prolongs Lifespan

Marisa M. Merino,[1] Christa Rhiner,[1] Jesus M. Lopez-Gay,[1,2] David Buechel,[1] Barbara Hauert,[1] and Eduardo Moreno[1,*]
[1]Institute of Cell Biology, IZB, University of Bern, Bern 3012, Switzerland
[2]Polarity Division and Morphogenesis, Institut Curie, CNRS UMR 3215, INSERM U934 Paris, France
*Correspondence: emoreno@izb.unibe.ch
http://dx.doi.org/10.1016/j.cell.2014.12.017
This is an open access article under the CC BY license (http://creativecommons.org/licenses/by/3.0/).

SUMMARY

Viable yet damaged cells can accumulate during development and aging. Although eliminating those cells may benefit organ function, identification of this less fit cell population remains challenging. Previously, we identified a molecular mechanism, based on "fitness fingerprints" displayed on cell membranes, which allows direct fitness comparison among cells in *Drosophila*. Here, we study the physiological consequences of efficient cell selection for the whole organism. We find that fitness-based cell culling is naturally used to maintain tissue health, delay aging, and extend lifespan in *Drosophila*. We identify a gene, *azot*, which ensures the elimination of less fit cells. Lack of *azot* increases morphological malformations and susceptibility to random mutations and accelerates tissue degeneration. On the contrary, improving the efficiency of cell selection is beneficial for tissue health and extends lifespan.

INTRODUCTION

Individual cells can suffer insults that affect their normal functioning, a situation often aggravated by exposure to external damaging agents. A fraction of damaged cells will critically lose their ability to live, but a different subset of cells may be more difficult to identify and eliminate: viable but suboptimal cells that, if unnoticed, may adversely affect the whole organism (Moskalev et al., 2013).

What is the evidence that viable but damaged cells accumulate within tissues? The somatic mutation theory of aging (Kennedy et al., 2012; Szilard, 1959) proposes that over time cells suffer insults that affect their fitness, for example, diminishing their proliferation and growth rates, or forming deficient structures and connections. This creates increasingly heterogeneous and dysfunctional cell populations disturbing tissue and organ function (Moskalev et al., 2013). Once organ function falls below a critical threshold, the individual dies. The theory is supported by the experimental finding that clonal mosaicism occurs at unexpectedly high frequency in human tissues as a function of time, not only in adults due to aging (Jacobs et al., 2012; Laurie et al., 2012), but also in human embryos (Vanneste et al., 2009).

Does the high prevalence of mosaicism in our tissues mean that it is impossible to recognize and eliminate cells with subtle mutations and that suboptimal cells are bound to accumulate within organs? Or, on the contrary, can animal bodies identify and get rid of unfit viable cells?

One indirect mode through which suboptimal cells could be eliminated is proposed by the "trophic theory" (Levi-Montalcini, 1987; Moreno, 2014; Raff, 1992; Simi and Ibáñez, 2010), which suggested that Darwinian-like competition among cells for limiting amounts of survival-promoting factors will lead to removal of less fit cells. However, it is apparent from recent work that trophic theories are not sufficient to explain fitness-based cell selection, because there are direct mechanisms that allow cells to exchange "cell-fitness" information at the local multicellular level (Moreno and Rhiner, 2014).

In *Drosophila*, cells can compare their fitness using different isoforms of the transmembrane protein Flower. The "fitness fingerprints" are therefore defined as combinations of Flower isoforms present at the cell membrane that reveal optimal or reduced fitness (Merino et al., 2013; Rhiner et al., 2010). The isoforms that indicate reduced fitness have been called Flower^{Lose} isoforms, because they are expressed in cells marked to be eliminated by apoptosis called "Loser cells" (Rhiner et al., 2010). However, the presence of Flower^{Lose} isoforms at the cell membrane of a particular cell does not imply that the cell will be culled, because at least two other parameters are taken into account: (1) the levels of Flower^{Lose} isoforms in neighboring cells: if neighboring cells have similar levels of Lose isoforms, no cell will be killed (Merino et al., 2013; Rhiner et al., 2010); (2) the levels of a secreted protein called Sparc, the homolog of the Sparc/Osteonectin protein family, which counteracts the action of the Lose isoforms (Portela et al., 2010).

Remarkably, the levels of Flower isoforms and Sparc can be altered by various insults in several cell types, including: (1) the appearance of slowly proliferating cells due to partial loss of ribosomal proteins, a phenomenon known as cell competition (Baillon and Basler, 2014; de Beco et al., 2012; Hogan et al., 2011; Morata and Ripoll, 1975; Moreno et al., 2002; Tamori and Deng, 2011); (2) the interaction between cells with slightly higher levels of d-Myc and normal cells, a process termed supercompetition (de la Cova et al., 2004; Moreno and Basler, 2004); (3) mutations in signal transduction pathways like Dpp signaling (Portela et al., 2010; Rhiner et al., 2010); or (4) viable neurons forming part of incomplete ommatidia (Merino et al., 2013). Intriguingly, the role of Flower isoforms is cell type specific, because certain isoforms acting as Lose marks in epithelial cells

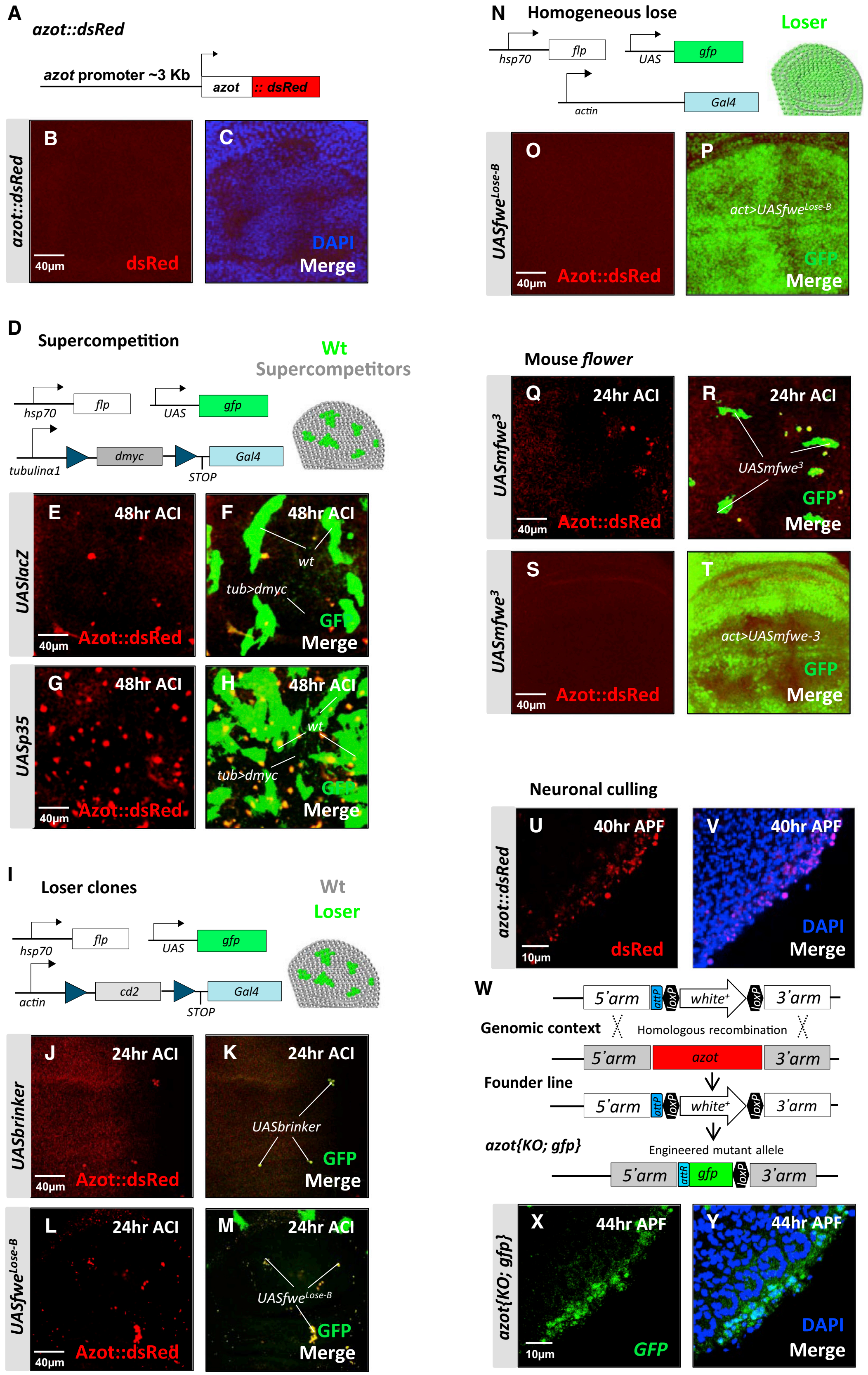

(legend on next page)

(Rhiner et al., 2010) are part of the fitness fingerprint of healthy neurons (Merino et al., 2013). Therefore, an exciting picture starts to appear, in which varying levels of Sparc and different isoforms of Flower are produced by many cell types, acting as direct molecular determinants of cell fitness.

Here, we aimed to clarify how cells integrate fitness information in order to identify and eliminate suboptimal cells. Subsequently, we analyzed what are the physiological consequences of efficient cell selection for the whole organism.

RESULTS

Azot Is Expressed in Cells Undergoing Negative Selection

In order to discover the molecular mechanisms underlying cell selection in *Drosophila*, we analyzed genes transcriptionally induced using an assay where WT cells (*tub>Gal4*) are outcompeted by dMyc-overexpressing supercompetitors (*tub>dmyc*) (Figure 1D) due to the increased fitness of these dMyc-overexpressing cells (Rhiner et al., 2010). The expression of *CG11165* (Figure S1A available online) was strongly induced 24 hr (hr) after the peak of *flower* and *sparc* expression (Figure S1B). In situ hybridization revealed that *CG11165* mRNA was specifically detected in Loser cells that were going to be eliminated from wing imaginal discs due to cell competition (Figure S1C). The gene, which we named *ahuizotl* (*azot*) after a multihanded Aztec creature selectively targeting fishing boats to protect lakes (Reeves, 2006), consists of one exon. *azot's* single exon encodes for a four EF-hand-containing cytoplasmic protein of the canonical family (Figures S1D and S1E) that is conserved, but uncharacterized, in multicellular animals (Figure S1A).

To monitor Azot expression, we designed a translational reporter resulting in the expression of Azot::dsRed under the control of the endogenous *azot* promoter in transgenic flies (Figure 1A). Azot expression was not detectable in most wing imaginal discs under physiological conditions in the absence of competition (Figures 1B and 1C). We next generated mosaic tissue of two clonal populations, which are known to trigger competitive interactions resulting in elimination of otherwise viable cells. Cells with lower fitness were created by confronting WT cells with dMyc-overexpressing cells (Figures 1E–1H) (Moreno and Basler, 2004), by downregulating Dpp signaling (Moreno et al., 2002) (Figures 1I–1K), by overexpressing Flower^{Lose} isoforms (Rhiner et al., 2010) (Figures 1L and 1M), in cells with reduced Wg signaling (Figure S1F) (Vincent et al., 2011), by suppressing Jak-Stat signaling (Rodrigues et al., 2012) in subgroups of cells (Figure S1G) or by generating *Minute* clones (Lolo et al., 2012; Morata and Ripoll, 1975; Simpson, 1979) (Figure S1H). Azot expression was not detectable in nonmosaic tissue of identical genotype (Figures 1N–1P; Figures S1I and S1J), nor in control clones overexpressing *UASlacZ* (Figure S1K). On the contrary, Azot was specifically activated in all tested scenarios of cell competition, specifically in the cells undergoing negative selection (''Loser cells'') (Figures 1D–1M). Azot expression was not repressed by the caspase inhibitor protein P35 (Figures 1G and 1H).

Because Flower proteins are conserved in mammals (Petrova et al., 2012), we decided to test if they are also able to regulate *azot*. Mouse Flower isoform 3 (mFlower^3) has been shown to act as a ''classical'' Lose isoform, driving cell elimination when expressed in scattered groups of cells (Petrova et al., 2012), a situation where *azot* was induced in Loser cells (Figures 1Q and 1R) but is not inducing cell selection when expressed ubiquitously a scenario where *azot* was not expressed (Figures 1S and 1T). This shows that the mouse Flower^{Lose} isoforms function in *Drosophila* similarly to their fly homologs.

Interestingly, *azot* is not a general apoptosis-activated gene because its expression is not induced upon *eiger*, *hid*, or *bax* activation, which trigger cell death (Fuchs and Steller, 2011; Gaumer et al., 2000) (Figures S1L–S1N). Azot was also not expressed during elimination of cells with defects in apicobasal polarity (Figure S1O) or undergoing epithelial exclusion-mediated apoptosis (*dCsk*) (Figures S1P and S1Q) (Vidal et al., 2006).

Next, we analyzed if *azot* is expressed during the elimination of peripheral photoreceptors in the pupal retina, a process mediated by Flower-encoded fitness fingerprints (Merino et al., 2013). Thirty-six to 38 hr after pupal formation (APF), when Flower$^{Lose\text{-}B}$ expression begins in peripheral neurons (Merino et al., 2013), we could not detect Azot expression in the peripheral edge (Figures S1R–S1U). At later time points (40 and 44 hr APF), Azot expression is visible and restricted to the peripheral edge where photoreceptor neurons are eliminated (Figures 1U and 1V). This expression was confirmed with another reporter line, *azot{KO; gfp}*, where *gfp* was directly inserted at the *azot* locus using genomic engineering techniques (Huang et al., 2009) (Figures 1W–1Y).

From these results, we conclude that Azot expression is activated in several contexts where suboptimal and viable cells are normally recognized and eliminated.

Azot Is Required to Eliminate Loser Cells and Unwanted Neurons

To understand Azot function in cell elimination, we generated *azot* knockout (KO) flies, whereby the entire *azot* gene was deleted (Figure 1W). Next, we analyzed Azot function using

Figure 1. Azot Is Expressed during Cell Selection of Viable Unfit Cells

(A–M) Expression analysis of Azot during different types of cell competition. For all pictures, Azot::dsRed reporter (A) is in red, and merges show outcompeted clones (green, marked with GFP) of several genotypes. DAPI is in blue. The following genotypes were analyzed: (B and C) *azot:dsRed* and (D–F) *tub>dmyc* background (black) and WT cells marked with GFP (green). Clones were generated as shown in (D) and analyzed 48 hr ACI. (G and H) *tub>dmyc* background (black) and WT cells marked with GFP (green) expressing in addition to the P35 caspase inhibitor (*UASp35*). Forty-eight hourr ACI. (I–M) Flip-out clones (green) generated as shown in (I) and overexpressing *brinker* (*UASbrinker)* (J and K), $fwe^{Lose\text{-}B}$ ($UASfwe^{Lose\text{-}B}$) (L and M), or $mfwe^3$ ($UASmfwe^3$).

(Q and R) Twenty-four hour ACI.

(N–P, S, and T) General overexpression of $UASfwe^{Lose\text{-}B}$ and $UASmfwe^3$ using the actin promoter as shown in (N).

(U–Y) Pupal retinas at different developmental time points. (U and V) Expression analysis of Azot (red), using Azot::dsRed, in peripheral photoreceptors at 40 hr after pupa formation (APF) (U and V). (W) Genomic engineering strategy used for the generation of *azot* knockout (KO) flies. (X and Y) GFP expression (green) driven by the *azot* promoter in *azot{KO; gfp}*, 44 hr APF, DAPI (blue, Y).

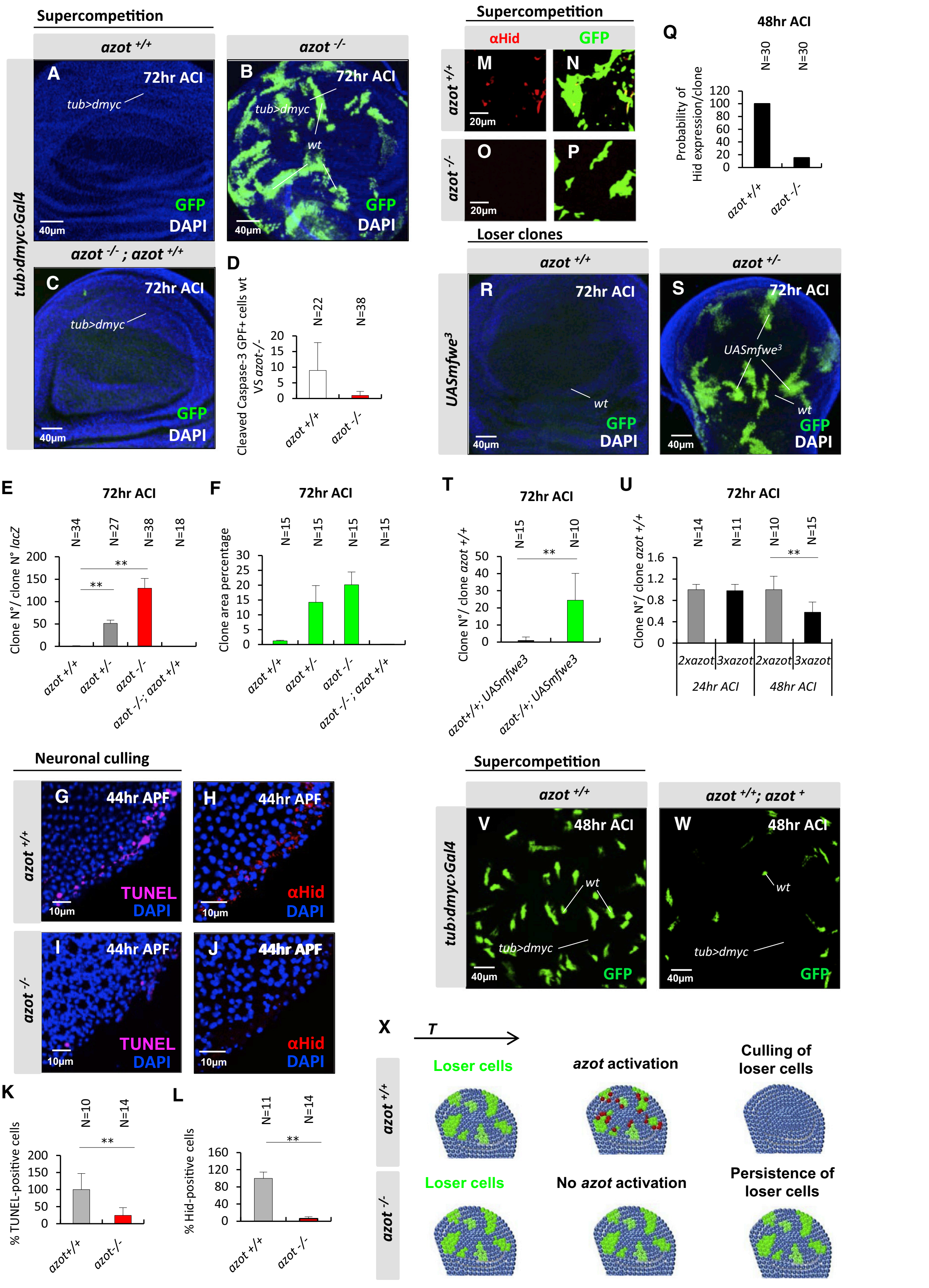

(legend on next page)

dmyc-induced competition. In the absence of Azot function, loser cells were no longer eliminated (Figures 2A–2F), showing a dramatic 100-fold increase in the number of surviving clones (Figures 2B and 2E). Loser cells occupied more than 20% of the tissue 72 hr after clone induction (ACI) (Figures 2B and 2F). Moreover, using *azot{KO; gfp}* homozygous flies (that express GFP under the *azot* promoter but lack Azot protein), we found that loser cells survived and showed accumulation of GFP (Figures S2A and S2B). From these results, we conclude that *azot* is expressed by loser cells and is essential for their elimination.

In addition, clone removal was delayed in an *azot* heterozygous background (50-fold increase, 15%) (Figures 2E and 2F), compared to control flies with normal levels of Azot (1-fold, 1%) (Figures 2A, 2E, and 2F). Cell elimination capacity was fully restored by crossing two copies of Azot::dsRed into the $azot^{-/-}$ background (0.5-fold, 0.2%) demonstrating the functionality of the fusion protein (Figures 2C, 2E, and 2F). Silencing *azot* with two different RNAis was similarly able to halt selection during *dmyc*-induced competition (Figures S2C–S2P). Next, in order to determine the role of Azot's EF hands, we generated and overexpressed a mutated isoform of Azot (Pm4Q12) carrying, in each EF hand, a point mutation known to abolish Ca^{2+} binding (Maune et al., 1992). Although overexpression of wild-type *azot* in negatively selected cells did not rescue the elimination (Figures S2E, S2I, S2L, and S2P), overexpression of the mutant AzotPm4Q12 reduced cell selection (Figures S2H, S2I, S2O, and S2P), functioning as a dominant-negative mutant. This shows that Ca^{2+} binding is important for Azot function. Finally, staining for apoptotic cells corroborated that the lack of Azot prevents cell elimination, because cell death was reduced 8-fold in mosaic epithelia containing loser cells (Figure 2D).

Next, we analyzed the role of *azot* in elimination of peripheral photoreceptor neurons in the pupal retina using homozygous *azot* KO flies (Figures 2G–2L). Pupal retinas undergoing photoreceptor culling (44 hr APF) of $azot^{+/+}$ and $azot^{-/-}$ flies were stained for the cell death marker TUNEL (Figures 2G and 2I) and the proapoptotic factor Hid (Figures 2H and 2J). Consistent with the expression pattern of Azot, the number of Hid and TUNEL-positive cells was dramatically decreased in $azot^{-/-}$ retinas (Figures 2I–2L) compared to $azot^{+/+}$ retinas (Figures 2G, 2H, 2K, and 2L).

Those results showed that Azot was required to induce cell death and Hid expression during neuronal culling. Therefore, we tested if that was also the case in the wing epithelia during *dmyc*-induced competition. We found that Hid was expressed in loser cells and that the expression was strongly reduced in the absence of Azot function (Figures 2M–2Q).

Finally, forced overexpression of $Flower^{Lose}$ isoforms from *Drosophila* (Figures S2Q, S2R, and S2T) and mice (Figures 2R–2T; Figures S2S and S2U) were unable to mediate WT cell elimination when Azot function was impaired by mutation or silenced by RNAi.

These results suggested that *azot* function was dose sensitive, because heterozygous *azot* mutant flies displayed delayed elimination of loser cells when compared with *azot* WT flies (Figure 2E). We therefore took advantage of our functional reporter Azot::dsRed (Figures 2C and 2E) to test whether cell elimination could be enhanced by increasing the number of genomic copies of *azot*. We found that tissues with three functional copies of *azot* were more efficient eliminating loser cells during *dmyc*-induced competition and most of the clones were culled 48 hr ACI (Figures 2U–2W).

From these results, we conclude that *azot* expression is required for the elimination of Loser cells and unwanted neurons (Figure 2X).

Azot Maintains Tissue Fitness during Development

Next, we asked what could be the consequences of decreased cell selection at the tissue and organismal level. To this end, we took advantage of the viability of homozygous *azot* KO flies. We observed an increase of several developmental aberrations. We focused on the wings, where cell competition is best studied and, because aberrations were easy to define, which comprised melanotic areas, blisters, and wing margin nicks (Figures 3A–3E). Wing defects of *azot* mutant flies could be rescued by introducing two copies of *azot::dsRed*, showing that the phenotypes are specifically caused by loss of Azot function (Figures 3A–3E).

Next, we reasoned that mild tissue stress should increase the need for fitness-based cell selection after damage. First, in order to generate multicellular tissues scattered with suboptimal cells, we exposed larvae to UV light (Figure 3F) and monitored Azot expression in wing discs of UV-irradiated WT larvae, which were stained for cleaved caspase-3, 24 hr after treatment (Figures 3G–3K). Under such conditions, Azot was found to be expressed in cleaved caspase-3-positive cells (Figures 3H–3K). All Azot-positive cells showed caspase activation and 17% of cleaved caspase-positive cells expressed Azot (Figure 3G). This suggested that Azot-expressing cells are culled from the tissue. To confirm this, we looked at later time points (3 days after

Figure 2. Azot Is Required to Eliminate Loser Cells and Unwanted Neurons

(A–F) Analysis of *azot* KO during *dmyc*-induced supercompetition 72 hr ACI. (D) Quantification of cleaved caspase-3 and GFP-positive cells during *dmyc*-induced supercompetition in $azot^{+/+}$ and $azot^{-/-}$ backgrounds ($p < 0.01$) 72 hr ACI. (E) Quantification of number of clones; the following backgrounds were analyzed: (A and E) $azot^{+/+}$, (E) $azot^{+/-}$ ($p < 0.01$), (B and E) $azot^{-/-}$ ($p < 0.01$), and (C and E) $azot^{-/-};^{+/+}$ ($p > 0.05$). (F) Percentage of the wing pouch occupied by the *wt* cells in the (A and F) $azot^{+/+}$, (F) $azot^{+/-}$, (B and F) $azot^{-/-}$, (C and F) $azot^{-/-};^{+/+}$.

(G–L) Role of *azot* during neuronal culling in the pupal retina. (K and L) Quantification of the number of apoptotic (TUNEL-positive, magenta) or Hid-expressing (red) peripheral photoreceptors, in $azot^{+/+}$ (G, H, K, and L) and $azot^{-/-}$ ($p < 0.01$) (I, J, K, and L) flies. DAPI is in blue.

(M–Q) Hid expression (red) in loser clones (green) during supercompetition 48 hr ACI in $azot^{+/+}$ (M, N, and Q) and $azot^{-/-}$ (O–Q) backgrounds.

(R–T) Seventy-two hour ACI $mfwe^3$-overexpressing clones ($UASmfwe^3$) in $azot^{+/+}$ (R and T) and $azot^{+/-}$ (S and T) backgrounds ($p < 0.01$).

(U–W) Analysis of an extra genomic copy of *azot* during *dmyc*-induced supercompetition. (U) Quantification of the number of clones during *dmyc*-induced supercompetition with or without an extra genomic copy of *azot*. (V and W) Discs analyzed 48 hr ACI in $azot^{+/+}$ (V) and $azot^{+/+}; azot^{+}$ ($p < 0.01$) (W).

(X) Azot expression is required for cell-competition-mediated apoptosis of loser cells. Data are represented as mean ± SEM.

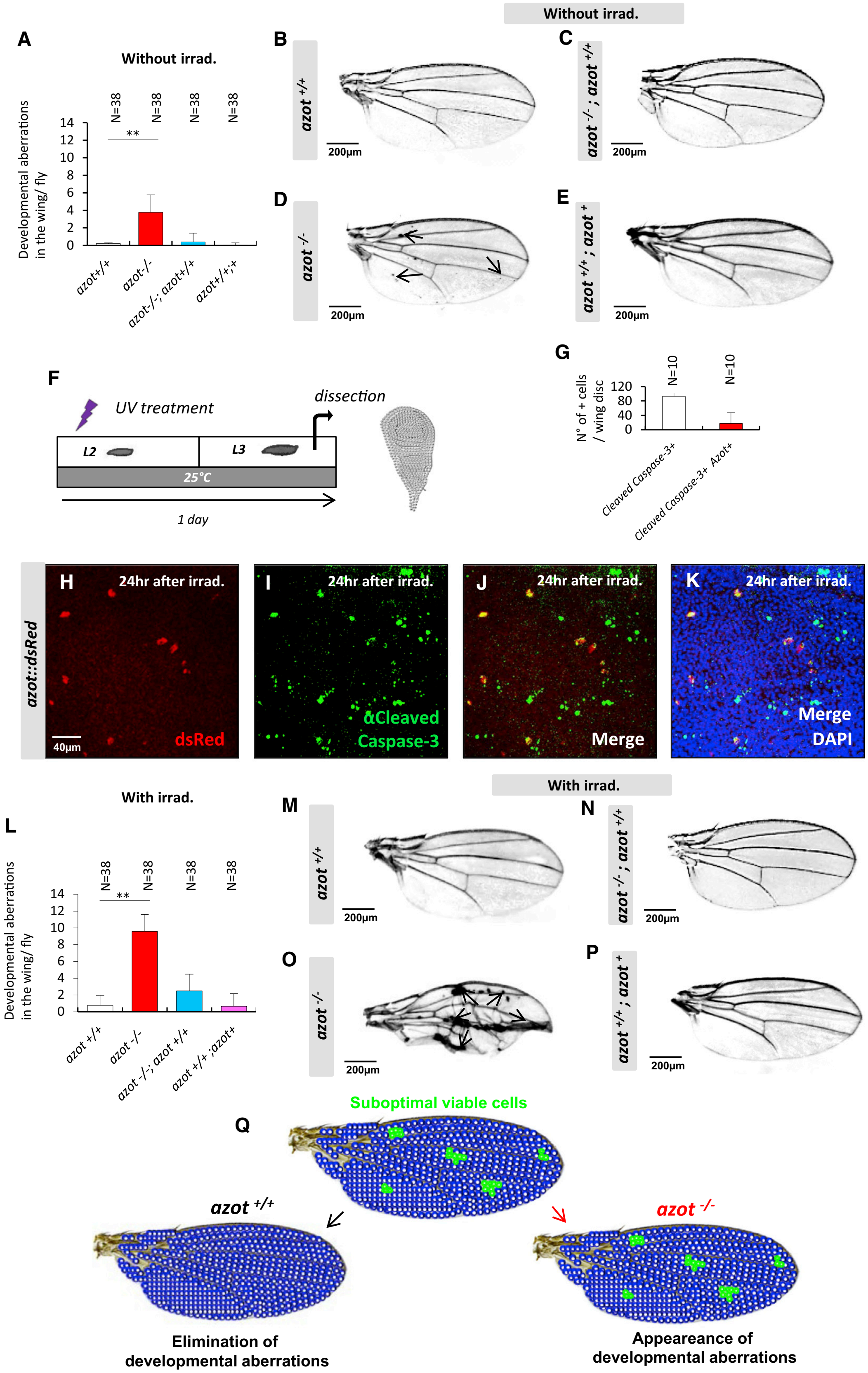

(legend on next page)

irradiation; Figure S3A) and found that the increase in Azot-positive cells was no longer detectable (Figures S3B–S3D). The elimination of *azot*-expressing cells after UV irradiation required *azot* function, because cells revealed by reporter *azot{KO; gfp}*, that express GFP instead of Azot, persisted in wing imaginal discs from *azot*-null larvae (Figures S3E–S3G). We therefore tested if lack of *azot* leads to a faster accumulation of tissue defects during organ development upon external damage. We irradiated $azot^{-/-}$ pupae 0 stage (Figures 3L–3P) and compared the number of morphological defects in adult wings to those in nonirradiated *azot* KO flies (Figures 3A–3E). We found that aberrations increased more than 2-fold when compared to nonirradiated $azot^{-/-}$ flies (Figures 3L–3P).

In order to functionally discriminate whether *azot* belongs to genes regulating apoptosis in general or is dedicated to fitness-based cell selection, we examined if *azot* silencing prevented Eiger/TNF-induced cell death (*GMR-Gal4,UASeiger*) (Figures S3H–S3N). Inhibiting apoptosis (*UASp35*) or *eiger* (*UASRNAieiger*) rescued eye ablation, whereas *azot* silencing and overexpression of AzotPm4Q12 did not (Figures S3I–S3N). Furthermore, *azot* silencing did not impair apoptosis during genitalia rotation (Figures S3O–S3R) (Suzanne et al., 2010) or cell death of epithelial precursors in the retina (Figures S3S–S3V) (Wolff and Ready, 1991).

The results showed above highlight the consequences of nonfunctional cell-quality control within developing tissues (Figure 3Q).

azot Promoter Computes Relative Flower^{Lose} and Sparc Levels

Next, we performed epistasis analyses to understand at which level *azot* is transcriptionally regulated. For this purpose, we again used the assay where WT cells are outcompeted by dMyc-overexpressing supercompetitors (Figure 1D). We have previously observed that *azot* induction is triggered upstream of caspase-3 activation and accumulated in outcompeted cells unable to die (Figures 1G and 1H). Then, we genetically modified upstream events of cell selection (Figures 4A–4G): silencing fwe^{Lose} transcripts by RNAi or overexpressing Sparc, both blocked the induction of Azot::dsRed in WT loser cells (Figures 4A–4D and 4G). In contrast, when outcompeted WT cells were additionally "weakened" by Sparc downregulation using RNAi, Azot is detected in almost all loser cells (Figures 4E–4G) compared to its more limited induction in the presence of endogenous Sparc (Figures 1E and 1F and 4G). Inhibiting JNK signaling with *UASpuc* (Martín-Blanco et al., 1998; Moreno et al., 2002) did not suppress Azot expression (Figures S4A and S4B).

Next, we analyzed the activation of Azot upon irradiation. Strikingly, we found that all Azot expression after irradiation was eliminated when Flower Lose was silenced and also when relative differences of Flower Lose where diminished by overexpressing high levels of Lose isoforms ubiquitously (Figures 4H–4K; Figure S4C). On the contrary, Azot was not suppressed after irradiation by expressing the prosurvival factor Bcl-2 or a p53 dominant negative (Brodsky et al., 2000; Gaumer et al., 2000) (Figures S4C–S4G). Those results show that Azot expression during competition and upon irradiation requires differences in Flower Lose relative levels.

Finally, we analyzed the regulation of Azot expression in neurons. Silencing *fwe* transcripts by RNAi blocked the induction of Azot::dsRed in peripheral photoreceptors (Figures 4L and 4M; Figure S4H). Because Wingless signaling induces Flower$^{Lose\text{-}B}$ expression in peripheral photoreceptors (Merino et al., 2013), we tested if overexpression of Daxin, a negative regulator of the pathway (Willert et al., 1999), affected Azot levels and found that it completely inhibited Azot expression (Figures S4H–S4J). Similarly, overexpression of the cell competition inhibitor Sparc also fully blocked Azot endogenous expression in the retina (Figures S4H, S4K, and S4L). Finally, ectopic overexpression of Flower$^{Lose\text{-}B}$ in scattered cells of the retina was sufficient to trigger ectopic Azot activation (Figures S4M–S4O). Those results show that photoreceptor cells also can monitor the levels of Sparc and the relative levels of Flower$^{Lose\text{-}B}$ before triggering Azot expression (Figure S4P).

The results described above suggest that the *azot* promoter integrates fitness information from neighboring cells, acting as a relative "cell-fitness checkpoint" (Figures 4N–4Q).

Cell Selection Is Active during Adulthood

To test if fitness-based cell selection is a mechanism active not only during development, but also during adult stages, we exposed WT adult flies to UV light and monitored Azot and Flower expression in adult tissues (Figures 5A–5T). UV irradiation of adult flies triggered cytoplasmic Azot expression in several adult tissues including the gut (Figures 5B–5E; Figures S5A and S5B) (Lemaitre and Miguel-Aliaga, 2013) and the adult brain (Figures 5F–5J) (Fernández-Hernández et al., 2013). Likewise, UV irradiation of adult flies triggered Flower Lose expression in the gut (Figures 5K–5N) and in the brain (Figures 5O–5T). Irradiation-induced Azot expression was unaffected by Bcl-2 but was eliminated when Flower Lose was silenced or when relative differences of Flower Lose where diminished in the gut (Figures S5C–S5E) and in the adult brain (Figures S5F–S5H). This suggests that the process of cell selection is active throughout the life history of the animal. Further confirming this conclusion,

Figure 3. *Azot* Mutants Show Developmental Aberrations

(A–E) Wings of 10- to 13-day-old flies and quantification of developmental aberrations in the wing of each genotype, ** < 0.01. (A and B) $azot^{+/+}$, (A and C) $azot^{-/-};azot^{+/+}$, (A and D) $azot^{-/-}$ and (A and E) $azot^{+/+};azot^{+}$.

(F–K) Azot and cleaved caspase-3 expression upon UV irradiation (2×10^{-2} J irradiation dose during second instar larvae, treatment as shown in F). (G) Quantification of the percentage of Azot and cleaved caspase-3-expressing cells after UV irradiation. (H) Azot::dsRed expression after UV irradiation (red), (I) cleaved caspase-3 (green) after UV irradiation, (J) merge, and (K) merge with DAPI (blue).

(L–P) Quantification of developmental aberrations and images of wings from 10- to 13-day-old flies after UV treatment (2×10^{-2} J, pupae stage 0) of genotypes (L and M) $azot^{+/+}$, (L and N) $azot^{-/-};azot^{+/+}$, (L and O) $azot^{-/-}$, and (L and P) $azot^{+/+};azot^{+}$.

(Q) Scheme showing the requirement of *azot* function for preventing developmental aberrations. Data are represented as mean ± SEM.

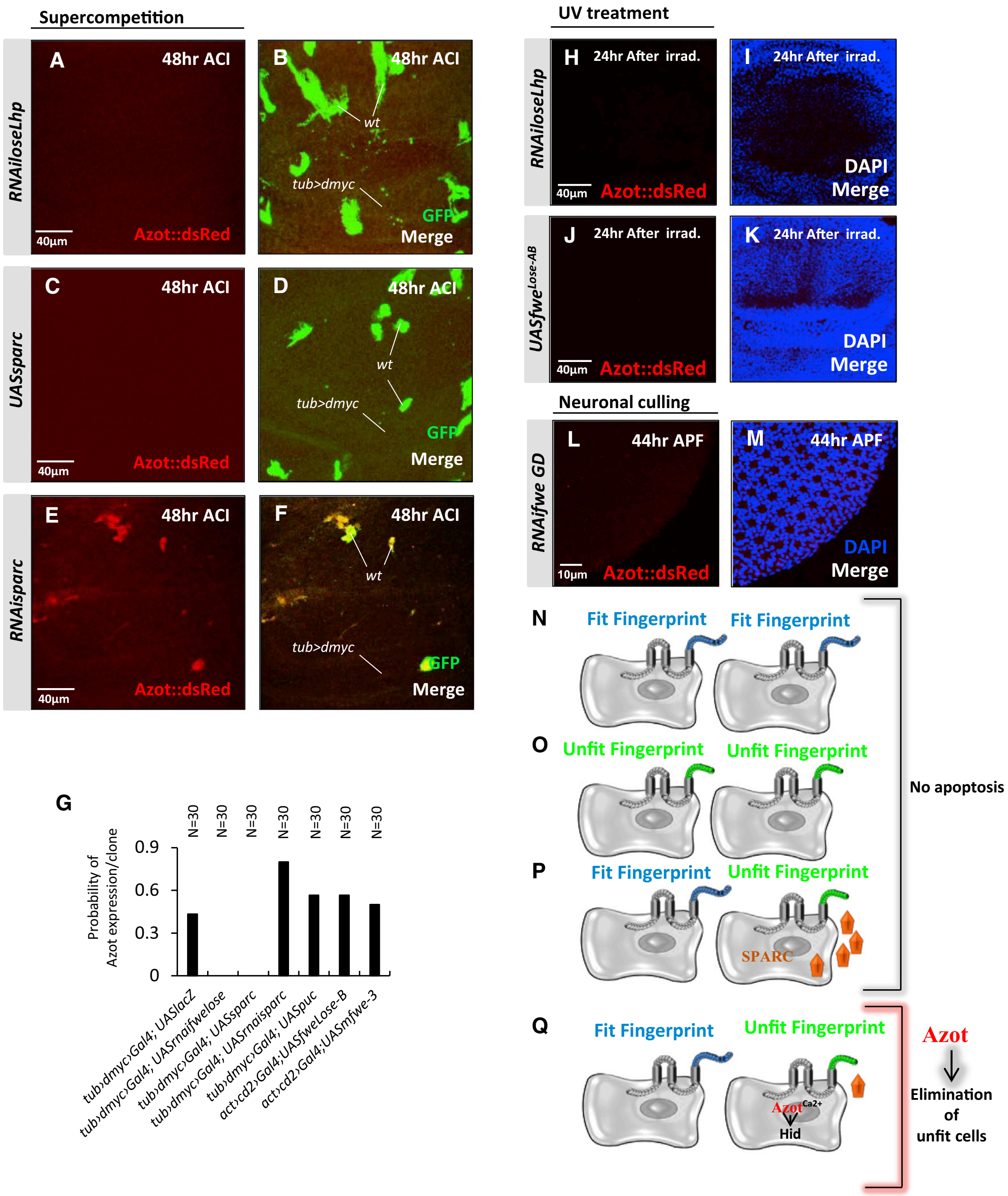

Figure 4. The *azot* Promoter Computes Relative Flower^{Lose} and Sparc Levels

(A–F) Epistasis analysis of the following genotypes during *dmyc*-induced supercompetition. (A and B) *UASRNAifwe*^{loseLhp}, (C and D) *UASsparc*, and (E and F) *UASRNAisparc*. Azot::dsRed is shown in red (A, C, and E) and merges with GFP in (B, D, and F).

(G) Graph showing the probability of finding Azot expression in a GFP marked clone in several genotypes.

(H–J) Azot::dsRed expression after UV irradiation (red) is suppressed when *UASRNAifwe*^{loseLhp} (H and I) or *UASfwe*$^{Lose\text{-}B}$ and *UASfwe*$^{Lose\text{-}A}$ (J and K) are expressed ubiquitously. Quantified in Figure S4C.

(L and M) Epistasis analysis of Azot expression in the *Drosophila* retina. Pupal retinas dissected 44 hr APF of *GMR-Gal4; RNAifwe (GD)*. Azot expression shown in red (L) and merge with nuclear marker DAPI in blue (M). Quantified in Figure S4H.

(N) Azot is not expressed in cells without Flower^{Lose} isoforms.

(O–Q) Cells expressing Flower^{Lose} but that are either surrounded by cells with equal or higher levels of Flower^{Lose} (O) or express high levels of Sparc (P) also do not activate *azot* expression. Cells with higher relative levels of Lose and not enough Sparc induce the expression of *azot* and are eliminated (Q).

Cell

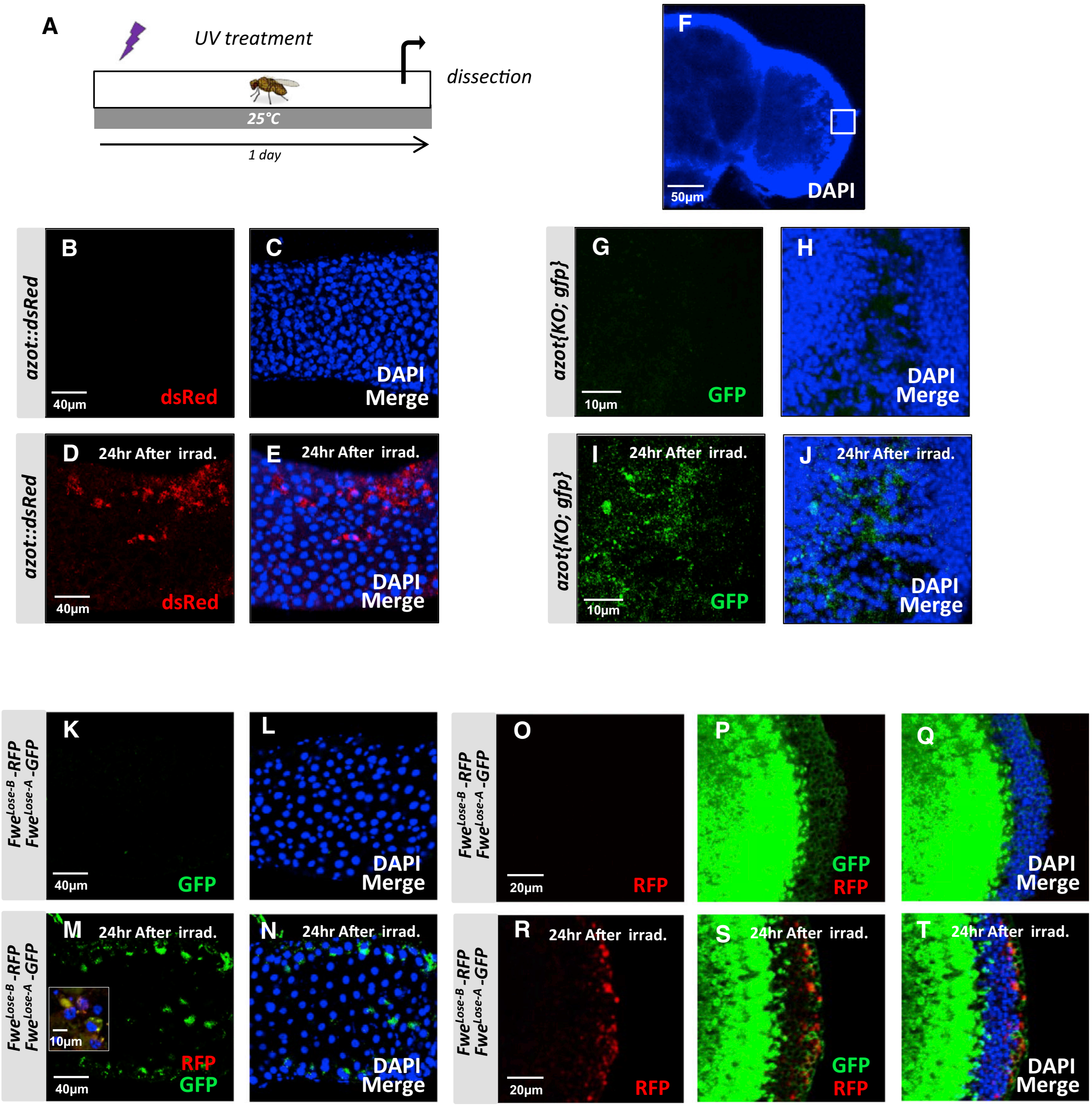

Figure 5. Expression of Flower Isoforms and Azot in Adult Flies with and without UV Irradiation

(A–E) Expression analysis of Azot (red, B and D) in the midgut without (B and C) and with (D and E) UV-irradiation treatment (as shown in A); (C) and (E) show merges with DAPI.

(F–J) Expression analysis of Azot using reporter line *azot{KO; gfp}* in the adult brain without (G and H) and after (I and J) UV-irradiation treatment merges with DAPI in (H and J).

(K–T) Expression analysis of Flower Lose isoforms Lose A (green) and Lose B (red) (*flower Lose-A-GFP*, *flower Lose-B-RFP*). (K and M) In the midgut without (K and L) and with (M and N) UV-irradiation treatment. (L and N) merges with DAPI. Inset in (M) shows $Fwe^{Lose\text{-}A}$ and $Fwe^{Lose\text{-}B}$ expression at higher magnification. (O–T) Expression of Flower Lose isoforms in the adult brain without (O–Q) and after (R–T) UV irradiation, merges with DAPI in (Q and T).

Azot function was essential for survival after irradiation, because more than 99% of *azot* mutant adults died 6 days after irradiation, whereas only 62.4% of WT flies died after the same treatment (Figure S5I). The percentage of survival correlated with the dose of *azot* because adults with three functional copies of *azot* had higher median survival and maximum lifespan than WT flies, or null mutant flies rescued with two functional *azot* transgenes (Figure S5J).

Those results show that in adult tissues external damage can induce cell-fitness deficits.

Role of Cell Selection during Aging

Lack of cell selection could affect the whole organism by two nonexclusive mechanisms. First, the failure to detect precancerous cells, which could lead to cancer formation and death of the individual. Second, the time-dependent accumulation of unfit

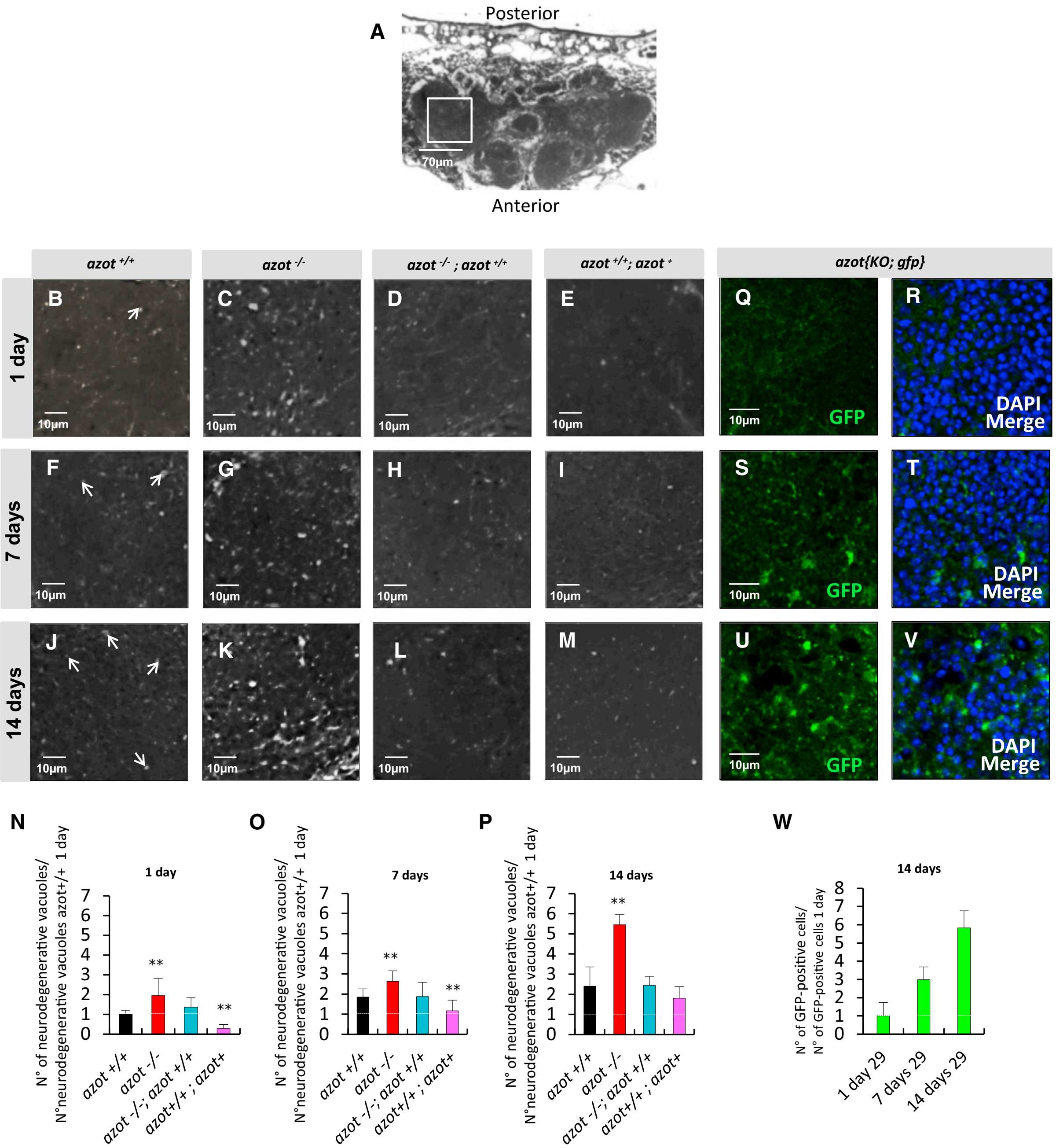

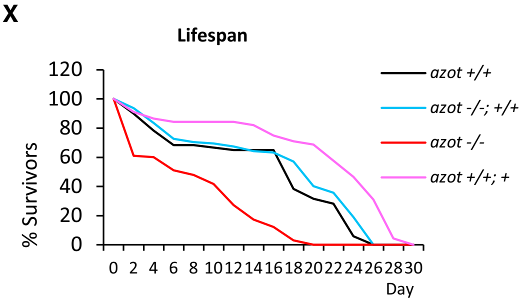

Y

Lifespan analysis 29°C

Genotype	Median survival (d)	% Median survival difference	Max. lifespan	% Max. lifespan difference	Log-Rank p
azot +/+	16.3	0%	24	0%	-
azot -/-; +/+	18.2	12%	24	0%	>0.05
azot -/-	7.8	-52.14%	18	-25%	<0.001
azot +/+; +	25.1	54%	28	17%	<0.001

(legend on next page)

but viable cells could lead to accelerated tissue and organ decay. We therefore tested both hypotheses.

It has been previously shown that cells with reduced levels for cell polarity genes like *scrib* or *dlg* are eliminated but can give rise to tumors when surviving (Igaki et al., 2009; Parisi et al., 2014; Tamori et al., 2010). We therefore checked if *azot* functions as a tumor suppressing mechanism in those cells (Figures S6A–S6M). Elimination of *dlg* and *scrib* mutant cells was not affected by RNAi against *azot* (Figures S6D–S6M) or when Azot function was impaired by mutation (Figures S6N–S6R), in agreement with the absence of *azot* induction in these mutant cells (Figures S1O and S6A–S6C). However, *azot* RNAi or the same *azot* mutant background efficiently rescued the elimination of clones with reduced Wg signaling (Vincent et al., 2011) (Figures S6J–S6M, S6Q, and S6R).

Moreover, the high number of suboptimal cells produced by UV treatment did not lead to tumoral growth in *azot*-null background (Figures S3E–S3G). Thus, tumor suppression mechanisms are not impaired in *azot* mutant backgrounds, and tumors are not more likely to arise in *azot*-null mutants.

Second, we tested whether the absence of *azot* accelerates tissue fitness decay in adult tissues. We focused on the adult brain, where neurodegenerative vacuoles develop over time and can be used as a marker of aging (Liu et al., 2012). We compared the number of vacuoles appearing in the brain of flies lacking *azot* ($azot^{-/-}$), WT flies ($azot^{+/+}$), flies with one extra genomic copy of the gene ($azot^{+/+}; azot^{+}$), and mutant flies rescued with two genomic copies of *azot* ($azot^{-/-};azot^{+/+}$). For all the genotypes analyzed, we observed a progressive increase in the number and size of vacuoles in the brain over time (Figures 6A–6P; Figure S6S). Interestingly, $azot^{-/-}$ brains showed higher number of vacuoles compared to control flies ($azot^{+/+}$ and $azot^{-/-};azot^{+/+}$) and a higher rate of vacuole accumulation developing over time (Figures 6N–6P). In the case of flies with three genomic copies of the gene ($azot^{+/+}; azot^{+}$), vacuole number tended to be the lowest (Figures 6E, 6I, and 6M–6P).

Next, we analyzed the cumulative expression of *azot* during aging of the adult brain. We detected positive cells as revealed by reporter *azot{KO; gfp}*, in homozygosis, that express GFP instead of Azot. We observed a time-dependent accumulation of *azot*-positive cells (Figures 6Q–6W).

From this, we conclude that *azot* is required to prevent tissue degeneration in the adult brain and lack of *azot* showed signs of accelerated aging. This suggested that *azot* could affect the longevity of adult flies (Figures 6X and 6Y). We found that flies lacking *azot* ($azot^{-/-}$) had a shortened lifespan with a median survival of 7.8 days, which represented a 52% decrease when compared to WT flies ($azot^{+/+}$), and a maximum lifespan of 18 days, 25% less than WT flies ($azot^{+/+}$). This effect on lifespan was *azot* dependent because it was completely rescued by introducing two functional copies of *azot* (Figures 6X and 6Y). On the contrary, flies with three functional copies of the gene ($azot^{+/+}; azot^{+}$) showed an increase in median survival and maximum lifespan of 54% and 17%, respectively.

In conclusion, *azot* is necessary and sufficient to slow down aging, and active selection of viable cells is critical for a long lifespan in multicellular animals.

Death of Unfit Cells Is Sufficient and Required for Multicellular Fitness Maintenance

Our results show the genetic mechanism through which cell selection mediates elimination of suboptimal but viable cells. However, using flip-out clones and MARCM (Lee and Luo, 2001), we found that Azot overexpression was not sufficient to induce cell death in wing imaginal discs (Figures S6T–S6Y). Because Hid is downstream of Azot, we wondered whether expressing Hid under the control of the *azot* regulatory regions could substitute for Azot function.

In order to test this hypothesis, we replaced the whole endogenous *azot* protein-coding sequence by the cDNA of the proapoptotic gene *hid* (*azot*{*KO; hid*} flies; see Figure 7A). In a second strategy, the whole endogenous *azot* protein-coding sequence was replaced by the cDNA of transcription factor *Gal4*, so that the *azot* promoter can activate any *UAS* driven transgene (*azot*{*KO; Gal4*} flies (Figure 7B). We then compared the number of morphological aberrations in the adult wings of six genotypes: first, homozygous *azot*{*KO; Gal4*} flies that lacked Azot; second, *azot*{*KO; hid*} homozygous flies that express Hid with the *azot* pattern in complete absence of Azot; third, $azot^{+/+}$ WT flies as a control; and finally three genotypes where the *azot*{*KO; Gal4*} flies were crossed with *UAShid*, *UASsickle*, another proapoptotic gene (Srinivasula et al., 2002), or *UASp35*, an apoptosis inhibitor. In the case of *UASsickle* flies, we introduced a second *azot* mutation to eliminate *azot* function. Interestingly, the number of morphological aberrations was brought back to WT levels in all the situations where the *azot* promoter was driving proapoptotic genes (*azot*{*KO; hid*}, *azot*{*KO; Gal4*} × *UAShid, azot*{*KO; Gal4*} × *UASsickle*, see Figures 7A–7J) with or without irradiation. On the contrary, expressing *p35* with the *azot* promoter was sufficient to produce morphological aberrations despite the presence of one

Figure 6. *azot* Is Required to Prevent Tissue Degeneration in the Adult Brain and to Promote Lifespan

(A–P) Brain integrity studies over time. (A) Axial plane of *Drosophila* WT brain counterstained with toluidine blue. (B–M) Magnification images of the central brain, counterstained with toluidine blue, showing degenerative vacuoles (white dots) of the following four genotypes over time: (1) $azot^{+/+}$, (2) $azot^{-/-}$, (3) $azot^{-/-}$; $azot^{+/+}$, and (4) $azot^{+/+}; azot^{+}$. (N–P) Number of neurodegenerative vacuoles. (N) Number of degenerative vacuoles per brain area (70 × 70 μm) after 1 day at 29°C ($azot^{+/+}$ n = 14, $azot^{-/-}$ [$p < 0.01$] n = 8, $azot^{-/-};azot^{+/+}$ n = 16 and $azot^{+/+}; azot^{+}$ [$p < 0.01$] n = 11). (O) Number of degenerative vacuoles per brain area after 7 days at 29°C ($azot^{+/+}$ n = 16, $azot^{-/-}$ [$p < 0.01$] n = 16, $azot^{-/-};azot^{+/+}$ n = 7 and $azot^{+/+}; azot^{+}$ [$p < 0.01$] n = 20). (P) Number of degenerative vacuoles per brain area after 14 days at 29°C ($azot^{+/+}$ n = 7, $azot^{-/-}$ [$p < 0.01$] n = 3, $azot^{-/-};azot^{+/+}$ n = 10 and $azot^{+/+}; azot^{+}$ n = 7).

(Q–V) Azot-positive cells (green, GFP) in *azot{KO; gfp}* homozygous flies after 1 day (Q and R), 7 days (S and T), and 14 days (U and V) at 29°C. DAPI is in blue.

(W) Number of Azot-positive cells per brain area (50 × 50 μm) in *azot{KO; gfp}* homozygous flies after 1 day (n = 11), 7 days (n = 15), and 14 days (n = 18) at 29°C.

(X) Lifespan studies of the same four genotypes at 29°C.

(Y) Lifespan values, including median survival and maximum lifespan, for the four genotypes.

Data are represented as mean ± SEM.

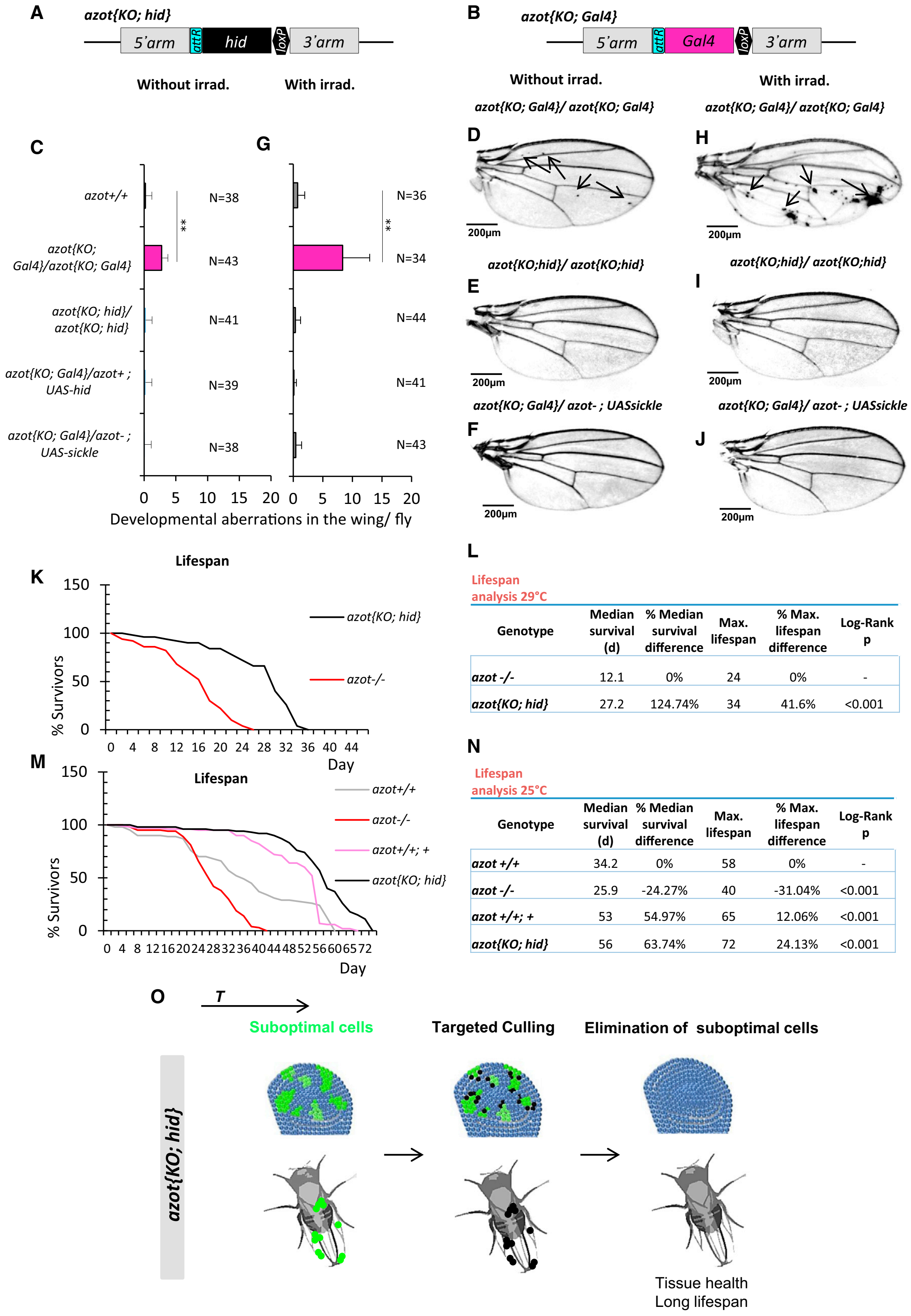

Lifespan analysis 29°C

Genotype	Median survival (d)	% Median survival difference	Max. lifespan	% Max. lifespan difference	Log-Rank p
azot -/-	12.1	0%	24	0%	-
azot{KO; hid}	27.2	124.74%	34	41.6%	<0.001

Lifespan analysis 25°C

Genotype	Median survival (d)	% Median survival difference	Max. lifespan	% Max. lifespan difference	Log-Rank p
azot +/+	34.2	0%	58	0%	-
azot -/-	25.9	-24.27%	40	-31.04%	<0.001
azot +/+; +	53	54.97%	65	12.06%	<0.001
azot{KO; hid}	56	63.74%	72	24.13%	<0.001

(legend on next page)

functional copy of *azot* (Figures S7A–S7H). Likewise, *p35*-expressing flies (*azot{KO; Gal4}/azot^{+}; UASp35*) did not survive UV treatments (Figure S7I), whereas a percentage of the flies expressing *hid* (26%) or *sickle* (28%) in *azot*-positive cells were able to survive (Figure S7I).

From this, we conclude that specifically killing those cells selected by the *azot* promoter is sufficient and required to prevent morphological malformations and provide resistance to UV irradiation.

Death of Unfit Cells Extends Lifespan

Next, we checked if the shortened longevity observed in *azot*$^{-/-}$ flies could be also rescued by killing *azot*-expressing cells with *hid* in the absence of Azot protein. We found that *azot {KO; hid}* homozygous flies had dramatically improved lifespan with a median survival of 27 days at 29°C, which represented a 125% increase when compared to *azot*$^{-/-}$ flies, and a maximum lifespan of 34 days, 41% more than mutant flies (Figures 7K and 7L).

Similar results were obtained at 25°C (Figures 7M and 7N). We found that flies lacking *azot* (*azot*$^{-/-}$) had a shortened lifespan with a median survival of 25 days, which represented a 24% decrease when compared to WT flies (*azot*$^{+/+}$), and a maximum lifespan of 40 days, 31% less than WT flies (*azot*$^{+/+}$). On the contrary, flies with three functional copies of the gene (*azot*$^{+/+}$*; azot*$^{+}$) or flies where *azot* is replaced by *hid* (*azot{KO; hid}* homozygous flies) showed an increase in median survival of 54% and 63% and maximum lifespan of 12% and 24%, respectively.

Finally, we tested the effects of dietary restriction on longevity of those flies (Partridge et al., 2005) (Figures S7J and S7K). We found that dietary restriction could extend both the median survival and the maximum lifespan of all genotypes (Figures S7J and S7K). Interestingly, dietary restricted flies with three copies of the gene *azot* showed a further increase in maximum lifespan of 35% (Figure S7K). This shows that dietary restriction and elimination of unfit cells can be combined to maximize lifespan.

In conclusion, eliminating unfit cells is sufficient to increase longevity, showing that cell selection is critical for a long lifespan in *Drosophila*.

DISCUSSION

Here, we show that active elimination of unfit cells is required to maintain tissue health during development and adulthood. We identify a gene *(azot)*, whose expression is confined to suboptimal or misspecified but morphologically normal and viable cells. When tissues become scattered with suboptimal cells, lack of *azot* increases morphological malformations and susceptibility to random mutations and accelerates age-dependent tissue degeneration. On the contrary, experimental stimulation of *azot* function is beneficial for tissue health and extends lifespan. Therefore, elimination of less fit cells fulfils the criteria for a hallmark of aging (López-Otín et al., 2013).

Although cancer and aging can both be considered consequences of cellular damage (Greaves and Maley, 2012; López-Otín et al., 2013), we did not find evidence for fitness-based cell selection having a role as a tumor suppressor in *Drosophila*. Our results rather support that accumulation of unfit cells affect organ integrity and that, once organ function falls below a critical threshold, the individual dies.

We find Azot expression in a wide range of "less fit" cells, such as WT cells challenged by the presence of "supercompetitors," slow proliferating cells confronted with normal proliferating cells, cells with mutations in several signaling pathways (i.e., Wingless, JAK/STAT, Dpp), or photoreceptor neurons forming incomplete ommatidia. In order to be expressed specifically in "less fit" cells, the transcriptional regulation of *azot* integrates fitness information from at least three levels: (1) the cell's own levels of Flower^{Lose} isoforms, (2) the levels of Sparc, and (3) the levels of Lose isoforms in neighboring cells. Therefore, Azot ON/OFF regulation acts as a cell-fitness checkpoint deciding which viable cells are eliminated. We propose that by implementing a cell-fitness checkpoint, multicellular communities became more robust and less sensitive to several mutations that create viable but potentially harmful cells. Moreover, *azot* is not involved in other types of apoptosis, suggesting a dedicated function, and—given the evolutionary conservation of Azot—pointing to the existence of central cell selection pathways in multicellular animals.

EXPERIMENTAL PROCEDURES

In Situ Hybridization

We followed the protocol described in Rhiner et al. (2010). Probe sequences are available upon request.

Drosophila Genetics

Stocks and crosses were kept at 25°C in standard media. The following stocks were used: ywf;tub > dmyc > Gal4/Cyo;UASgfp; azot::dsRed/TM6B; GMR-Gal4; azot::dsRed/TM6B; ywf;tub > dmyc > Gal4,azot^{-}/Cyo;UASgfp; ywf;tub

Figure 7. Culling Azot-Expressing Cells Is Sufficient and Required for Multicellular Fitness Maintenance

(A and B) Knockin (KI) schemes (A) *azot{KO; Gal4}* and (B) *azot{KO;hid}*.

(C–F) Wings from 10- to 13-day-old flies and quantification of developmental aberrations of the following five genotypes: (C) *azot*$^{+/+}$, (C and D) *azot{KO; Gal4}/azot {KO; Gal4}*, (C and E) *azot{KO;hid}/azot{KO;hid}*, (C and F) *azot{KO; Gal4}/azot*$^{-}$ *;UASsickle*, and (C) *azot{KO; Gal4}/azot*$^{+}$ *;UAShid*.

(G–J) Wings from 10- to 13-day-old flies and quantification of developmental aberrations after UV irradiation of the same five genotypes. Irradiation dose of 2×10^{-2} J administered during pupal stage 0.

(K and L) Comparative lifespan studies of genotypes *azot{KO;hid}/azot{KO;hid}* and *azot*$^{-/-}$ at 29°C.

(L) Median and maximum survival of genotypes *azot{KO;hid}/azot{KO;hid}* and *azot*$^{-/-}$.

(M and N) Lifespan studies at 25°C of the following four genotypes: (1) *azot*$^{+/+}$, (2) *azot*$^{-/-}$, (3) *azot*$^{+/+}$*; azot*$^{+}$, and (4) *azot{KO;hid}/azot{KO;hid}*. (N) Median and maximum survival of the four genotypes.

(O) Scheme showing that specifically killing Azot-expressing cells with the general proapoptotic factor Hid is sufficient to prevent morphological malformations and rescue *azot* mutant phenotypes.

Data are represented as mean ± SEM.

> dmyc > Gal4,azot^{-}/Cyo;UASrfp; ywf;act > y+ > gal4,azot^{-}/Cyo;UASgfp; ywf;act > y+ > Gal4/Cyo;UASRNAiazot; azot{KO;gfp}; azot{KO;hid}; azot{KO; Gal4}; UASbrk;act > cd2 > Gal4,UASgfp/TM6B; act > y+ > Gal4,UASgfp;azot::dsRed/TM6B; w;flowerUbi-YFP,flowerLose-A-GFP,flowerLose-B-RFP; ywf; Ubigfp,MinuteFRT42/Cyo; ywf;FRT42/Cyo; hsFlp,UAS-CD8-GFP;GAL80 FRT 40A/Cyo;tub > G4/TM6B; ywFlp;armZFRT40A/Cyo;MKRS/TM6B; ywf;patched-Gal4; apterous-Gal4; GMR-Gal4,UASeiger; RNAifwe^{Loselhp} (Merino et al., 2013); ywf;UASmfwe^{3}; ywf;UASsparc/TM6B; UASfwe$^{Lose\text{-}B}$; UASfwe$^{Lose\text{-}A}$; UASfwe$^{Lose\text{-}A}$,UASfwe$^{Lose\text{-}B}$; UASp35; UASpuckered; UASdAxin/TM3; UAShid; UASsickle; UASbax; UASbcl2; UASp53DN; UASRNAifweGD; UASRNAisparc(16678); UASRNAiazotGD(18166); UASRNAiazotKK(102353); UASRNAiscribble(Bloomington); UASRNAidlg(Bloomington); UASRNAihopscotch(Bloomington); UASRNAieigerGD; ywf;Cyo/if;UASazot/TM6B; ywf;Cyo/if;UASazot-HA/TM6B; ywf;Cyo/if;UASazotpm4Q12/TM6B; ywf; UASlacZ; and UASCSK-IR.

Clone Induction

Flip-out clones were generated after heat shock at 37°C between 5 and 15 min. For ubiquitous expression experiments larvae were subjected to 45 min heat shock for all cells to perform flip-out and activate Gal4 under the control of the *actin* promoter (*act>Gal4*).

Azot Reporter: azot::dsRed

The genomic region 3 kb upstream plus the full exon was cloned in pRedStinger vector using XbaI and KpnI restriction sites. Primer sequences are available upon request.

Overexpressing Constructs

cDNA of *azot* was fully sequenced and subcloned into the pUASattB vector using XbaI and KpnI restriction sites. In order to generate N- and C-terminal HA-tagged forms, the respective cDNAs were amplified with primers containing the HA sequence and subcloned into KpnI and XbaI sites of pUASattB. Primer sequences are available upon request.

Azotpm4Q12

Site-directed mutagenesis was used to create point mutations that changed glutamic acid (E) to glutamine (Q) as shown in Figure S1A. Primer sequences are available upon request.

Azot Knockout Generation

We followed the genomic engineering strategy described in Huang et al. (2009); homologous regions are shown in (Figure 1A). Primer sequences are available upon request.

Knockin Generation

Knockout founder line (Figure 2A) was used for the generation of knockin flies as described in Huang et al. (2009). cDNA of *gfp*, *hid*, and *Gal4* was used for the generation of *azot{KO; gfp}*, *azot{KO; hid}*, and *azot{KO; Gal4}* knockin lines. Primer sequences are available upon request.

Immunohistochemistry

Standard immunohistochemistry protocol was used for antibody detection (Rhiner et al., 2010). For the generation of specific antibodies against Azot, N-terminal peptide MEDISHEERVLILDTFR was used to immunize rabbits. Anti-Wingless (ms, 1:50) was from DSHB, anti-caspase-3 (rabbit, 1:100) was from Cell Signaling Technology, anti-KDEL (rabbit, 1;100) was from Abcam, anti-cytochrome *c* (mouse, 1:800) was from BD Pharmingen, anti-Hid (rabbit, 1:50) and anti-HA (rat, 1:250) were from Roche, and anti-βGal (mouse, 1:200) was from Promega. TUNEL staining performed as described (Lolo et al., 2012). Confocal images acquired with Leica SP2 and SP5 microscopes.

UV Treatments

Treatments were performed using a UV Stratalinker 2400 machine (UV-B 254 nm). Adult flies were subjected to 2×10^{-2} J dose of UV irradiation when they were 1–3 days old and analyzed for Azot and Flower isoform expression 24 hr later. For lifespan experiments after irradiation, a dose of 5×10^{-2} J was used. Larvae and pupae were subjected to 2×10^{-2} J dose of UV irradiation, and Azot expression or developmental aberrations were analyzed.

Longevity Assays

Cohorts of 100 female flies (1–3 days old) of the same genetic background were collected and kept at 29°C or 25°C on standard food (3.4 l water, 280 g maize, 36 g agar, 120 g yeast, 300 g sugar syrup, 32 g potassium, 6 g methyl, 20 ml propionic acid). Surviving flies were counted every 2 days (He and Jasper, 2014).

Dietary Restriction Assays

Cohorts of 100 female flies (1–3 days old) were collected and kept at 29°C on water-diluted standard food (one to one). Surviving flies were counted every 2 days.

Brain Studies

Brain Integrity

Adult flies kept at 29°C of the selected time points and genotypes were analyzed for the appearance of neurodegenerative vacuoles over time in the central brain as previously described (Kretzschmar et al., 1997).

Azot Expression

Adult flies *azot{KO; gfp}/azot{KO; gfp}* were kept at 29°C. The selected time points were analyzed for the number of GFP-positive cells in the central brain.

Statistical Analysis

For the rescue assay using azot KO in supercompetition (Figure 2E), rescue assay in supercompetition with azot RNAi and overexpression of the protein (Figures S2J–S2P), the rescue assay of clones with apicobasal defects and the clones with deficient Wg signaling (Figures S6N–S6R), and brain integrity studies over time (Figures 6A–6P), the data were analyzed with the K independent samples test. The post hoc DMS test was then used to detect significant differences.

For the caspase-positive cells in azot$^{+/+}$ and azot$^{-/-}$ background (Figure 2D), the rescue assay in overexpression of Flower^{lose} isoforms (Figures 2R–2T; Figure S2T), and azot overexpression in clones (Figures S6T–S6Y), all data were analyzed with two independent samples test (Mann-Whitney U test). Levene test was used to analyze number of cleaved caspase-3-positive cells, rescue assay of Flower^{Lose} isoforms, and number of azot-overexpressing clones.

For the quantification of the number of developmental aberrations before and after irradiation treatment in azot$^{+/+}$, azot$^{+/-}$, and azot$^{-/-}$, and azot$^{-/-}$; azot$^{+/+}$ background (Figures 3A–3E, 3L–3P, 7C–7J, and S7A–S7H), data were analyzed with the K independent samples test (Levene), and Levy-Tukey was used for post hoc analyses.

In the rescue assay in supercompetition using RNAi (24 hr ACI) (Figures S2C–S2I), the data were analyzed with ANOVA test.

In the quantification of eye size in apoptosis assay (Figures S3H–S3N), the data were analyzed with ANOVA. Bonferroni post hoc test was used to detect significant differences among genotypes.

For the functional assays of azot in retinas (Figures 2G–2L), azot dose sensitive (Figures 2U–2W), rescue assay in overexpression of mouse flower^{3} isoform (Figure S2U), and rescue assay of clones with apicobasal defects, and clones with deficient Wg signaling by azot RNAi (Figures S6D–S6M), all data were analyzed with Student's t test.

For the lifespan analysis (Figures 6X, 7K, 7M, and S7J), the log-rank test was used to study significant differences among the genotypes.

SUPPLEMENTAL INFORMATION

Supplemental Information includes seven figures and can be found with this article online at http://dx.doi.org/10.1016/j.cell.2014.12.017.

ACKNOWLEDGMENTS

We thank H. Bellen, H. Stocker, M. Vidal, H. Ryoo, A. Bergmann, B. Mignotte, M. Portela, VDRC, and Hybridoma bank and Bloomington for flies and antibodies; CONSOLIDER consortium for injecting flies; and members of our laboratory for comments on the manuscript. Work in our laboratory is funded by

the European Research Council, Swiss National Science Foundation, Josef Steiner Cancer Research Foundation, the bilateral Japanese-Swiss ST, and Swiss Cancer League.

Received: June 20, 2014
Revised: September 26, 2014
Accepted: November 24, 2014
Published: January 15, 2015

REFERENCES

Baillon, L., and Basler, K. (2014). Reflections on cell competition. Semin. Cell Dev. Biol. *32*, 137–144.

Brodsky, M.H., Nordstrom, W., Tsang, G., Kwan, E., Rubin, G.M., and Abrams, J.M. (2000). Drosophila p53 binds a damage response element at the reaper locus. Cell *101*, 103–113.

de Beco, S., Ziosi, M., and Johnston, L.A. (2012). New frontiers in cell competition. Developmental dynamics *241*, 831–841.

de la Cova, C., Abril, M., Bellosta, P., Gallant, P., and Johnston, L.A. (2004). Drosophila myc regulates organ size by inducing cell competition. Cell *117*, 107–116.

Fernández-Hernández, I., Rhiner, C., and Moreno, E. (2013). Adult neurogenesis in Drosophila. Cell Rep. *3*, 1857–1865.

Fuchs, Y., and Steller, H. (2011). Programmed cell death in animal development and disease. Cell *147*, 742–758.

Gaumer, S., Guénal, I., Brun, S., Théodore, L., and Mignotte, B. (2000). Bcl-2 and Bax mammalian regulators of apoptosis are functional in Drosophila. Cell Death Differ. *7*, 804–814.

Greaves, M., and Maley, C.C. (2012). Clonal evolution in cancer. Nature *481*, 306–313.

He, Y., and Jasper, H. (2014). Studying aging in Drosophila. Methods *68*, 129–133.

Hogan, C., Kajita, M., Lawrenson, K., and Fujita, Y. (2011). Interactions between normal and transformed epithelial cells: their contributions to tumourigenesis. Int. J. Biochem. Cell Biol. *43*, 496–503.

Huang, J., Zhou, W., Dong, W., Watson, A.M., and Hong, Y. (2009). From the cover: directed, efficient, and versatile modifications of the Drosophila genome by genomic engineering. Proc. Natl. Acad. Sci. USA *106*, 8284–8289.

Igaki, T., Pastor-Pareja, J.C., Aonuma, H., Miura, M., and Xu, T. (2009). Intrinsic tumor suppression and epithelial maintenance by endocytic activation of Eiger/TNF signaling in Drosophila. Dev. Cell *16*, 458–465.

Jacobs, K.B., Yeager, M., Zhou, W., Wacholder, S., Wang, Z., Rodriguez-Santiago, B., Hutchinson, A., Deng, X., Liu, C., Horner, M.J., et al. (2012). Detectable clonal mosaicism and its relationship to aging and cancer. Nat. Genet. *44*, 651–658.

Kennedy, S.R., Loeb, L.A., and Herr, A.J. (2012). Somatic mutations in aging, cancer and neurodegeneration. Mech. Ageing Dev. *133*, 118–126.

Kretzschmar, D., Hasan, G., Sharma, S., Heisenberg, M., and Benzer, S. (1997). The swiss cheese mutant causes glial hyperwrapping and brain degeneration in Drosophila. The Journal of neuroscience *17*, 7425–7432.

Laurie, C.C., Laurie, C.A., Rice, K., Doheny, K.F., Zelnick, L.R., McHugh, C.P., Ling, H., Hetrick, K.N., Pugh, E.W., Amos, C., et al. (2012). Detectable clonal mosaicism from birth to old age and its relationship to cancer. Nat. Genet. *44*, 642–650.

Lee, T., and Luo, L. (2001). Mosaic analysis with a repressible cell marker (MARCM) for Drosophila neural development. Trends Neurosci. *24*, 251–254.

Lemaitre, B., and Miguel-Aliaga, I. (2013). The digestive tract of Drosophila melanogaster. Annu. Rev. Genet. *47*, 377–404.

Levi-Montalcini, R. (1987). The nerve growth factor 35 years later. Science *237*, 1154–1162.

Liu, N., Landreh, M., Cao, K., Abe, M., Hendriks, G.J., Kennerdell, J.R., Zhu, Y., Wang, L.S., and Bonini, N.M. (2012). The microRNA miR-34 modulates ageing and neurodegeneration in Drosophila. Nature *482*, 519–523.

Lolo, F.N., Casas-Tintó, S., and Moreno, E. (2012). Cell competition time line: winners kill losers, which are extruded and engulfed by hemocytes. Cell Rep. *2*, 526–539.

López-Otín, C., Blasco, M.A., Partridge, L., Serrano, M., and Kroemer, G. (2013). The hallmarks of aging. Cell *153*, 1194–1217.

Martín-Blanco, E., Gampel, A., Ring, J., Virdee, K., Kirov, N., Tolkovsky, A.M., and Martinez-Arias, A. (1998). puckered encodes a phosphatase that mediates a feedback loop regulating JNK activity during dorsal closure in Drosophila. Genes Dev. *12*, 557–570.

Maune, J.F., Klee, C.B., and Beckingham, K. (1992). Ca2+ binding and conformational change in two series of point mutations to the individual Ca(2+)-binding sites of calmodulin. J. Biol. Chem. *267*, 5286–5295.

Merino, M.M., Rhiner, C., Portela, M., and Moreno, E. (2013).). "Fitness fingerprints" mediate physiological culling of unwanted neurons in Drosophila. Curr. Biol. *23*, 1300–1309.

Morata, G., and Ripoll, P. (1975). Minutes: mutants of Drosophila autonomously affecting cell division rate. Dev. Biol. *42*, 211–221.

Moreno, E. (2014). Cancer: Darwinian tumour suppression. Nature *509*, 435–436.

Moreno, E., and Basler, K. (2004). dMyc transforms cells into super-competitors. Cell *117*, 117–129.

Moreno, E., and Rhiner, C. (2014). Darwin's multicellularity: from neurotrophic theories and cell competition to fitness fingerprints. Curr. Opin. Cell Biol. *31C*, 16–22.

Moreno, E., Basler, K., and Morata, G. (2002). Cells compete for decapentaplegic survival factor to prevent apoptosis in Drosophila wing development. Nature *416*, 755–759.

Moskalev, A.A., Shaposhnikov, M.V., Plyusnina, E.N., Zhavoronkov, A., Budovsky, A., Yanai, H., and Fraifeld, V.E. (2013). The role of DNA damage and repair in aging through the prism of Koch-like criteria. Ageing Res. Rev. *12*, 661–684.

Parisi, F., Stefanatos, R.K., Strathdee, K., Yu, Y., and Vidal, M. (2014). Transformed epithelia trigger non-tissue-autonomous tumor suppressor response by adipocytes via activation of Toll and Eiger/TNF signaling. Cell Rep. *6*, 855–867.

Partridge, L., Piper, M.D., and Mair, W. (2005). Dietary restriction in Drosophila. Mech. Ageing Dev. *126*, 938–950.

Petrova, E., López-Gay, J.M., Rhiner, C., and Moreno, E. (2012). Flower-deficient mice have reduced susceptibility to skin papilloma formation. Dis. Model. Mech. *5*, 553–561.

Portela, M., Casas-Tinto, S., Rhiner, C., López-Gay, J.M., Domínguez, O., Soldini, D., and Moreno, E. (2010). Drosophila SPARC is a self-protective signal expressed by loser cells during cell competition. Dev. Cell *19*, 562–573.

Raff, M.C. (1992). Social controls on cell survival and cell death. Nature *356*, 397–400.

Reeves, H.M. (2006). Sahagún's "Florentine codex," a little known Aztecan natural history of the Valley of Mexico. Arch. Nat. Hist. *33*, 302–321.

Rhiner, C., López-Gay, J.M., Soldini, D., Casas-Tinto, S., Martín, F.A., Lombardía, L., and Moreno, E. (2010). Flower forms an extracellular code that reveals the fitness of a cell to its neighbors in Drosophila. Dev. Cell *18*, 985–998.

Rodrigues, A.B., Zoranovic, T., Ayala-Camargo, A., Grewal, S., Reyes-Robles, T., Krasny, M., Wu, D.C., Johnston, L.A., and Bach, E.A. (2012). Activated STAT regulates growth and induces competitive interactions independently of Myc, Yorkie, Wingless and ribosome biogenesis. Development *139*, 4051–4061.

Simi, A., and Ibáñez, C.F. (2010). Assembly and activation of neurotrophic factor receptor complexes. Dev. Neurobiol. *70*, 323–331.

Simpson, P. (1979). Parameters of cell competition in the compartments of the wing disc of Drosophila. Dev. Biol. *69*, 182–193.

Srinivasula, S.M., Datta, P., Kobayashi, M., Wu, J.W., Fujioka, M., Hegde, R., Zhang, Z., Mukattash, R., Fernandes-Alnemri, T., Shi, Y., et al. (2002).). sickle,

a novel Drosophila death gene in the reaper/hid/grim region, encodes an IAP-inhibitory protein. Curr. Biol. *12*, 125–130.

Suzanne, M., Petzoldt, A.G., Speder, P., Coutelis, J.B., Steller, H., and Noselli, S. (2010).). Coupling of apoptosis and L/R patterning controls stepwise organ looping. Curr. Biol. *20*, 1773–1778.

Szilard, L. (1959). On the Nature of the Aging Process. Proc. Natl. Acad. Sci. USA *45*, 30–45.

Tamori, Y., and Deng, W.M. (2011). Cell competition and its implications for development and cancer. Journal of genetics and genomics *38*, 483–495.

Tamori, Y., Bialucha, C.U., Tian, A.G., Kajita, M., Huang, Y.C., Norman, M., Harrison, N., Poulton, J., Ivanovitch, K., Disch, L., et al. (2010). Involvement of Lgl and Mahjong/VprBP in cell competition. PLoS Biol. *8*, e1000422.

Vanneste, E., Voet, T., Le Caignec, C., Ampe, M., Konings, P., Melotte, C., Debrock, S., Amyere, M., Vikkula, M., Schuit, F., et al. (2009). Chromosome instability is common in human cleavage-stage embryos. Nat. Med. *15*, 577–583.

Vidal, M., Larson, D.E., and Cagan, R.L. (2006). Csk-deficient boundary cells are eliminated from normal Drosophila epithelia by exclusion, migration, and apoptosis. Dev. Cell *10*, 33–44.

Vincent, J.P., Kolahgar, G., Gagliardi, M., and Piddini, E. (2011). Steep differences in wingless signaling trigger Myc-independent competitive cell interactions. Dev. Cell *21*, 366–374.

Willert, K., Logan, C.Y., Arora, A., Fish, M., and Nusse, R. (1999). A Drosophila Axin homolog, Daxin, inhibits Wnt signaling. Development *126*, 4165–4173.

Wolff, T., and Ready, D.F. (1991). Cell death in normal and rough eye mutants of Drosophila. Development *113*, 825–839.

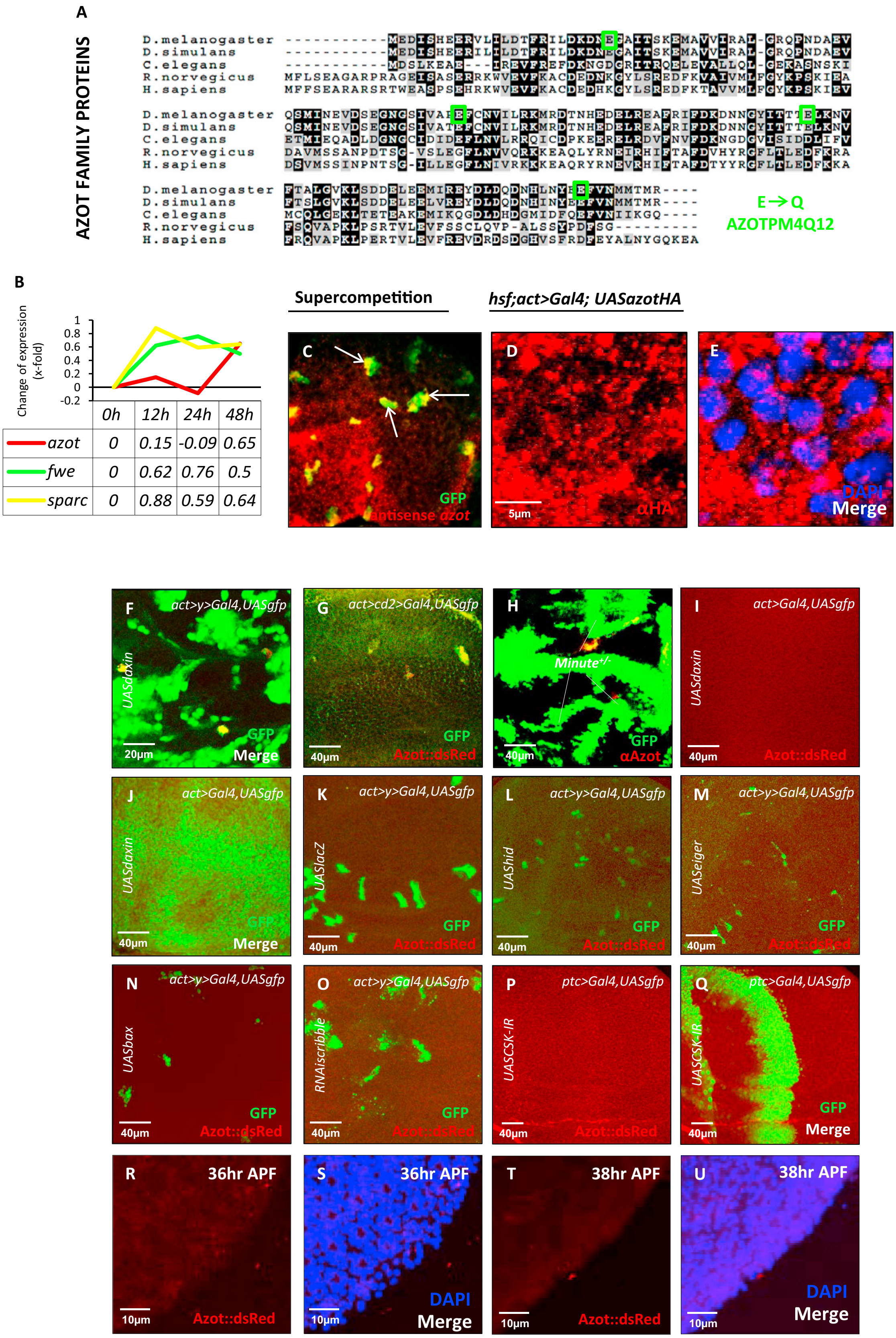

(legend on next page)

Figure S1. Azot Is Conserved throughout Evolution and Is Expressed in a Subset of Loser Cells in Cell Competition Scenarios, Related to Figure 1

(A) Alignment of Azot showing conservation in multicellular animals including humans. Point mutations highlighted for the generation of the pm4Q12 Azot mutant.

(B) Expression profile of different genes induced upon Supercompetition based on microarray data published in Rhiner et al., 2010.

(C) In situ analysis of *azot* RNA in *dmyc*-induced supercompetition, *azot* RNA probe (red), WT clones (green). Arrows show cells expressing *azot* RNA.

(D and E) HA-tagged Azot protein overexpressed in wing imaginal disc cells with *act-Gal4* driver is mainly cytoplasmic, anti-HA in red (D) and merged with DAPI (E).

(F–U) Expression analysis of Azot. Flip-out overexpressing clones of *UASdaxin* (Azot::dsRed, red) (F) and *RNAihopscotch* (Azot::dsRed, red) (G). (H) *Minute* heterozygous clones anti-Azot antibody (red). (I and J) Wing imaginal discs ubiquitously expressing Daxin and GFP (*act < Gal4; UASdaxin; UASgfp)*, (I) Azot::dsRed and merged with GFP (J). (K–O) Flip-out clones marked with GFP and overexpressing: (K) *UASlacZ*, (L) *UAShid*, (M) *UASeiger*, (N) *UASbax*, and (O) *RNAiscribble*. Azot expression revealed with Azot::dsRed from (K)–(O). (P and Q) *patched-Gal4; UASgfp; UASCSK-IR*, (red, Azot::dsRed). (R–U) Images of pupal retinas at different developmental time points. Expression analysis of Azot (red), using Azot::dsRed, in peripheral photoreceptors at different time points: 36hr after pupa formation (APF) (R and S) and 38hr APF (T and U).

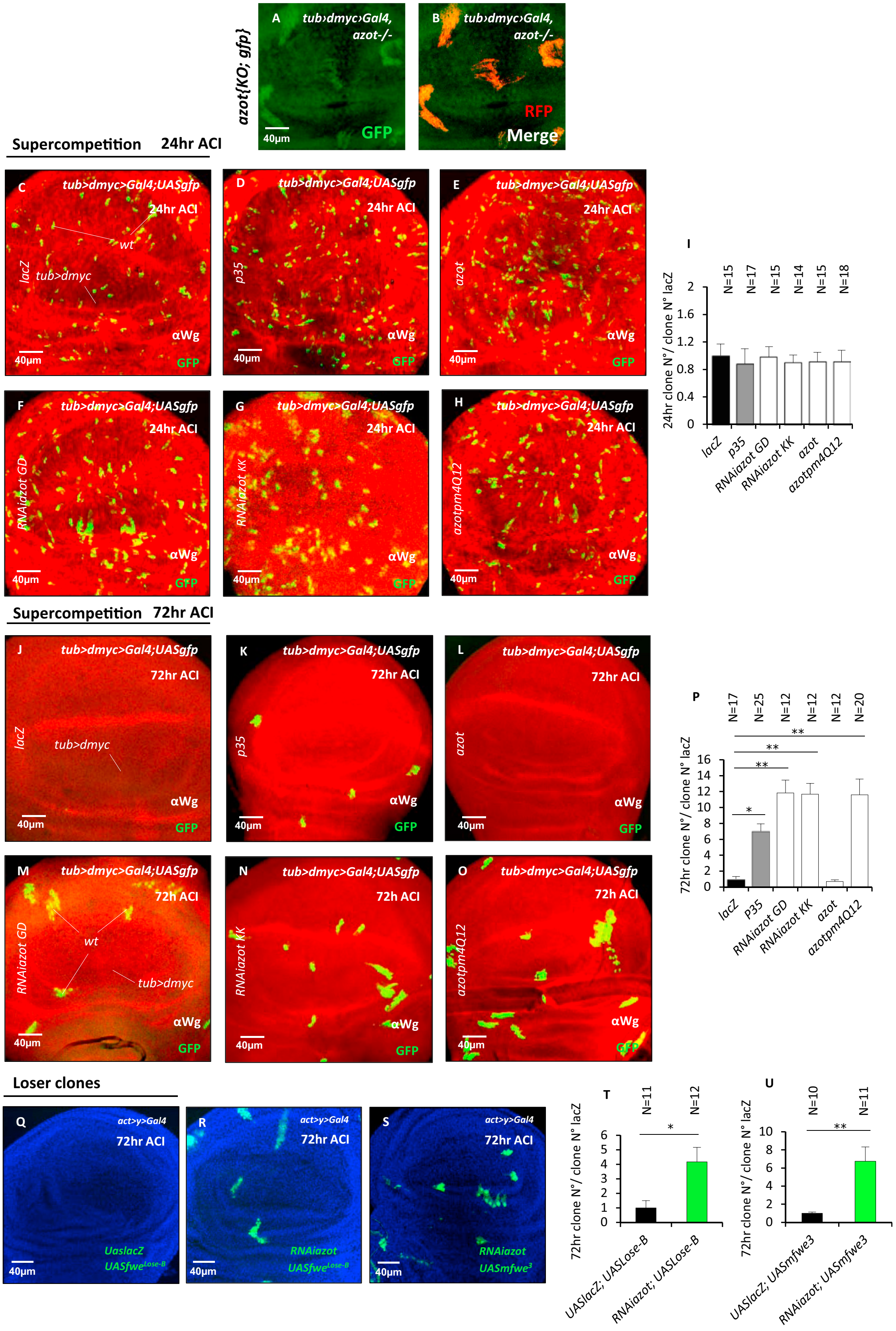

(legend on next page)

Figure S2. Azot Downregulation in Loser Cells during Cell Competition, Related to Figure 2

(A and B) *tub>dmyc* background (black) and WT cells marked with RFP (red) in *azot{KO; gfp}* homozygous background 72hr ACI.

(C–I) Images of wing imaginal discs 24hr ACI in *dmyc*-induced supercompetition of the following genotypes (C) *UASlacZ*, (D) *UASp35*, (E) *UASazot*, (F) *RNAiazot GD*, (G) *RNAiazot KK*, (H) *UASazotpm4Q12* (red, anti-Wingless), and quantification of number of clones at 24hr ACI (I).

(J–P) Quantification and images of WT clones in supercompetition of the following genotypes 72hr after ACI (anti-Wingless, red): (J) *UASlacZ*, (K) *UASp35* ($p < 0.05$), (L) *UASazot*, (M) *RNAiazot GD* ($p < 0.01$), (N) *RNAiazot KK* ($p < 0.01$), and (O) *UASazotpm4Q12* ($p < 0.01$) (red, anti-Wingless).

(Q–U) Flower Lose overexpressing clones (Q and T) *UASfwe*$^{Lose\text{-}B}$; *UASlacZ*, (R and T) *UASfwe*$^{Lose\text{-}B}$; *RNAiazot* ($p < 0.05$), (S and U) *UASmfwe*^{3}; *RNAiazot* ($p < 0.01$). *RNAiazot GD* line was used. Data are represented as mean ± SEM.

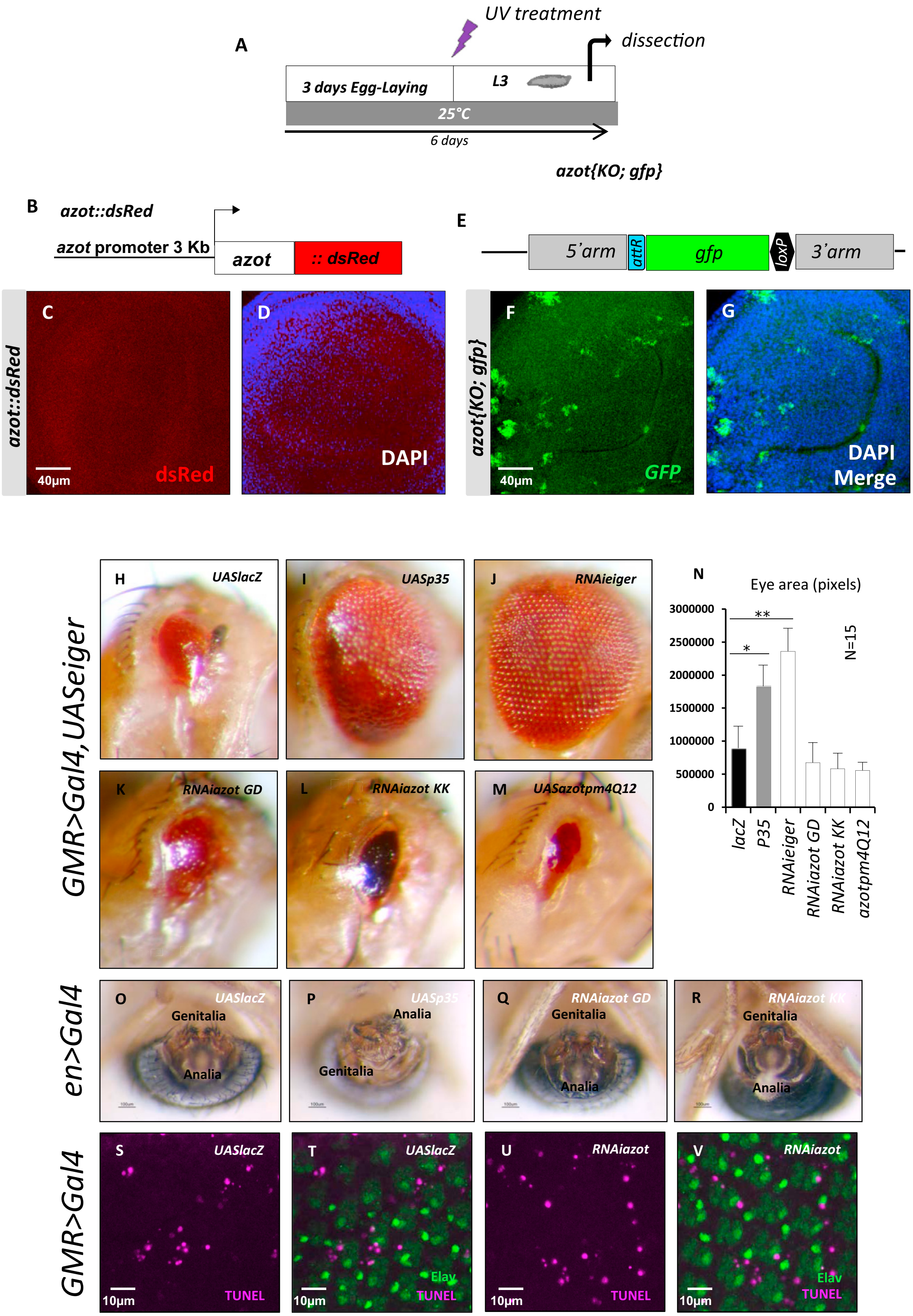

(legend on next page)

Figure S3. Azot Is Required to Eliminate a Subset of Cells after Irradiation but Is Not a General Proapoptotic Factor, Related to Figure 3

(A–G) Azot positive cells detected in wing imaginal discs after UV-treatment ($2x10^{-2}$J, 3 days after egg laying as shown in A). (B–D) Azot::dsRed functional reporter (B). Expression in the wing imaginal disc is shown in red (C) and merge with DAPI (blue, D). (E–G) *azot{KO; gfp}* reporter in homozygosis (E). Expression in the wing imaginal disc is shown in green (F) and merge with DAPI (blue, G).

(H–N) Images of *Drosophila* eyes and quantification of eye area (pixels), inducing apoptosis with *GMR-Gal4, UASeiger* in the following genotypes: (H) *UASlacZ*, (I) *UASp35*, (J) *RNAieiger*, (K) *RNAiazot GD*, (L) *RNAiazot KK*, (M) *UASazotpm4Q12* and quantification, (N) (* < 0.05 and ** < 0.01).

(O–R) Genitalia rotation assay, using *engrailed>Gal4* driver with the following genotypes: (O) *UASlacZ*, (P) *UASp35*, (Q) *RNAiazot GD*, (R) *RNAiazot KK*.

(S–V) images of *Drosophila* retina 24hr APF stained for TUNEL (magenta, S and U) and pan-neuronal marker Elav (green, T and V) of the following genotypes: (S and T) *GMR-Gal4; UASlacZ* and (U and V) *GMR-Gal4; RNAiazot*.

Data are represented as mean ± SEM.

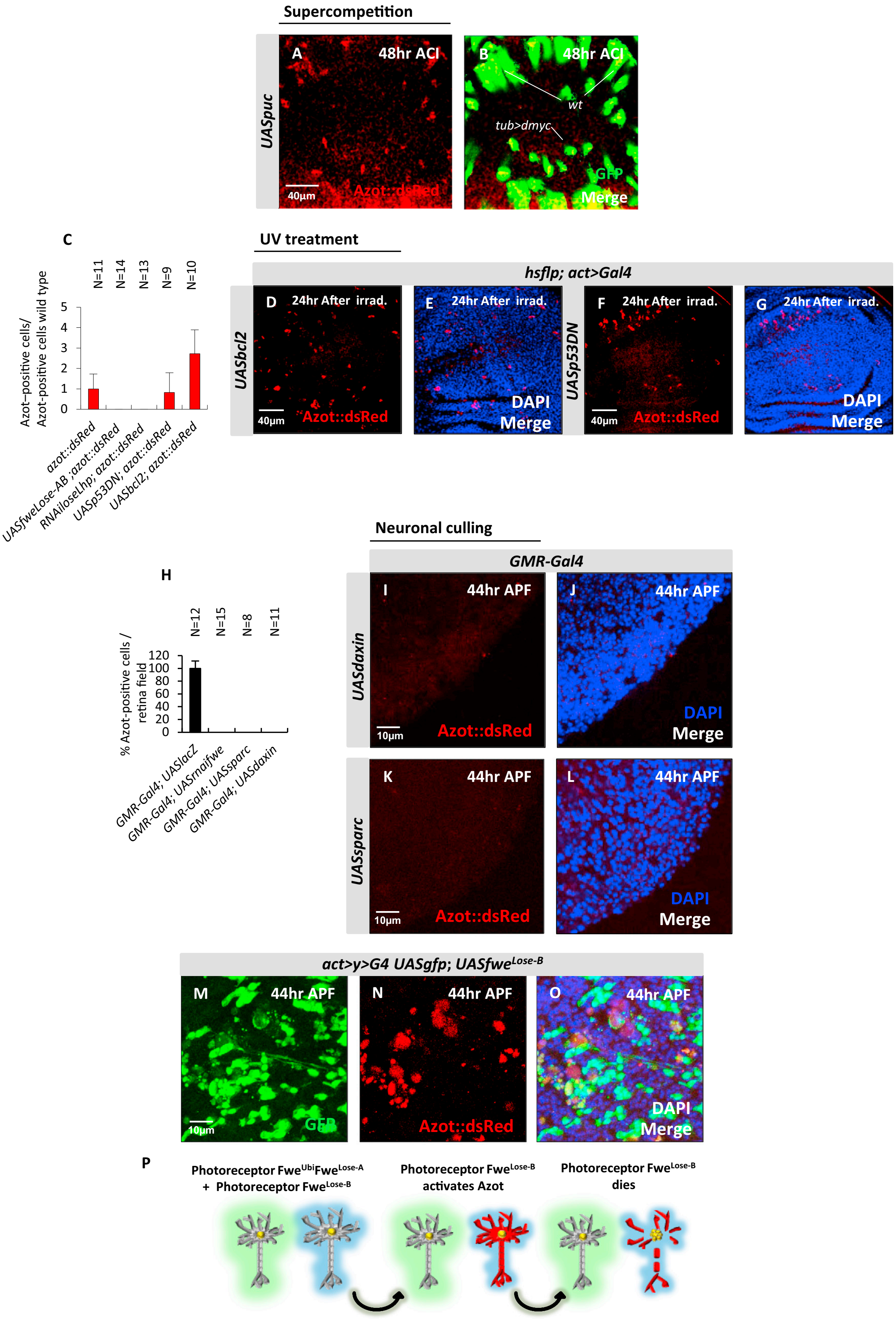

(legend on next page)

Figure S4. Regulation and Function of Azot, Related to Figure 4

(A and B) Epistasis analysis *UASpuc* during *dmyc* induced supercompetition Azot::dsRed is shown in red (A) and merges with GFP in (B).

(C–G) Azot::dsRed expression after UV-irradiation (red) is not suppressed when *UASbcl2* and *UASp53DN* are overexpressed ubiquitously with *actin-Gal4*. DAPI in blue.

(H–O) Quantification and epistasis analysis of *azot* in the retina. (H) Graph showing the probability of Azot expression in each genotype.

(I–L) Images of *Drosophila* retinas 44hr APF of the following genotypes: (I and J) *GMR-Gal4; UASdaxin* and (K and L) *GMR-Gal4; UASsparc*. Azot expression is shown in red (I and K) and merges with DAPI are shown in (J and L).

(M–O) *hsflp; act > y+STOP > Gal4, UASgfp;UASfwe*$^{Lose\text{-}B}$. GFP clones (green, M), Azot::dsRed (red, N) and merge with DAPI nuclear marker in (O).

(P) Scheme representing Azot-mediated elimination of peripheral photoreceptors.

Data are represented as mean ± SEM.

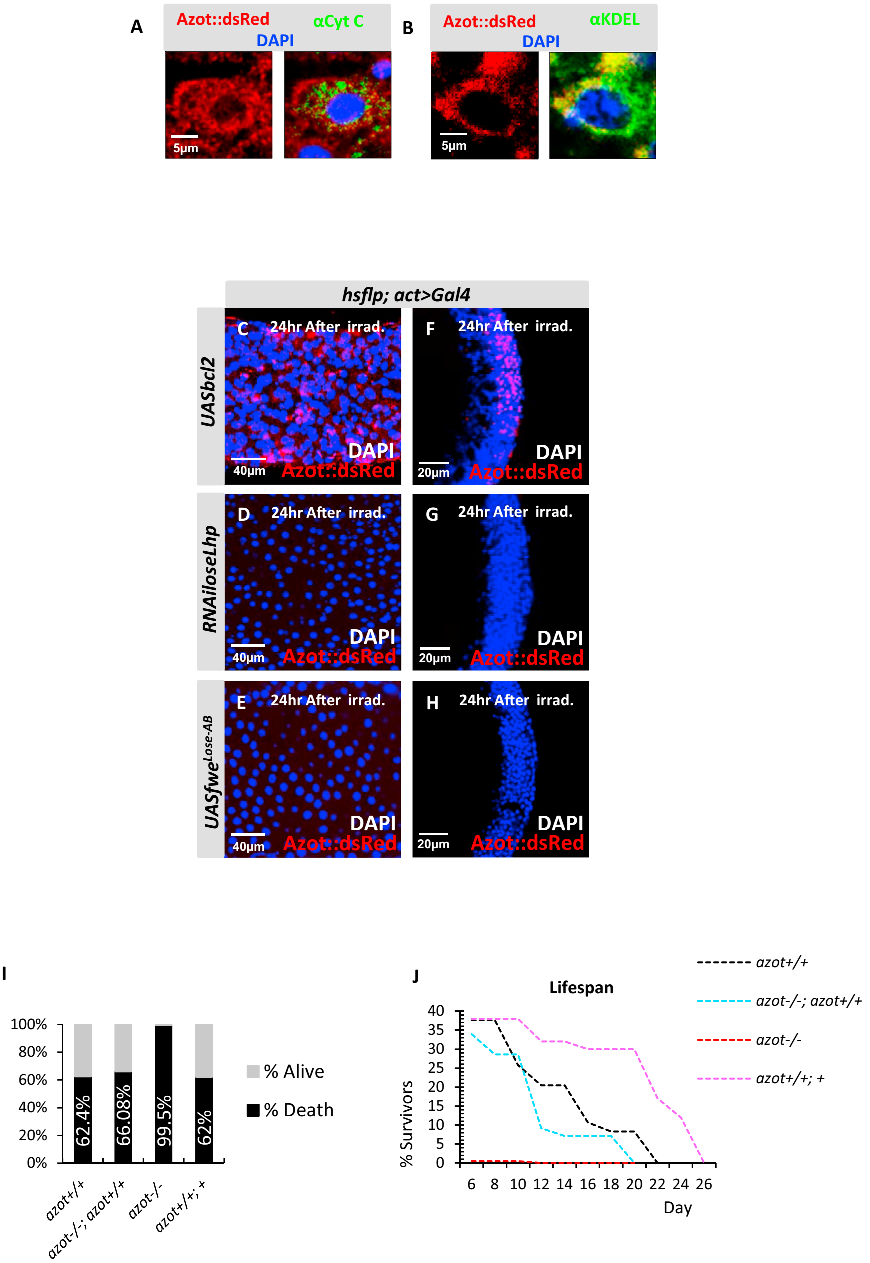

Figure S5. Expression and Function of Azot after UV Irradiation, Related to Figure 5

(A and B) Subcellular localization of Azot (red, Azot::dsRed) in enterocytes after irradiation co-stained with mitochondrial marker Cytochrome c (green, A) and ER marker KDEL (green, B). DAPI in blue. Azot does not co-localize with mitochondrial marker and partially co-localize with ER marker.

(C–H) Expression analyses of Azot (red, Azot::dsRed) after irradiation (2×10^{-2}J, 1-3 days old) in the midgut (C-E) and in the adult brain (F–H) using *actin-Gal4* to overexpress the following factors: (C and F) Bcl2 (*UASbcl2*), (D–G) *RNAi* against *flower lose* isoforms (UAS*RNAiloseLhp*) and (E–H) Flower LoseA and LoseB isoforms (*UASfwe*$^{Lose-A}$*, UASfwe*$^{Lose-B}$). Merges with DAPI (blue) and Azot (red).

(I) Percentage of adult survival 6 days post-irradiation (5×10^{-2}J, 1-3 days old) of the following 4 genotypes: 1) *azot*$^{+/+}$, 2) *azot*$^{-/-}$; *azot*$^{+/+}$, 3) *azot*$^{-/-}$, 4) *azot*$^{+/+}$*; azot*$^{+}$.

(J) Lifespan studies at 29°C 6 days after the same UV-irradiation treatment of the previous 4 genotypes.

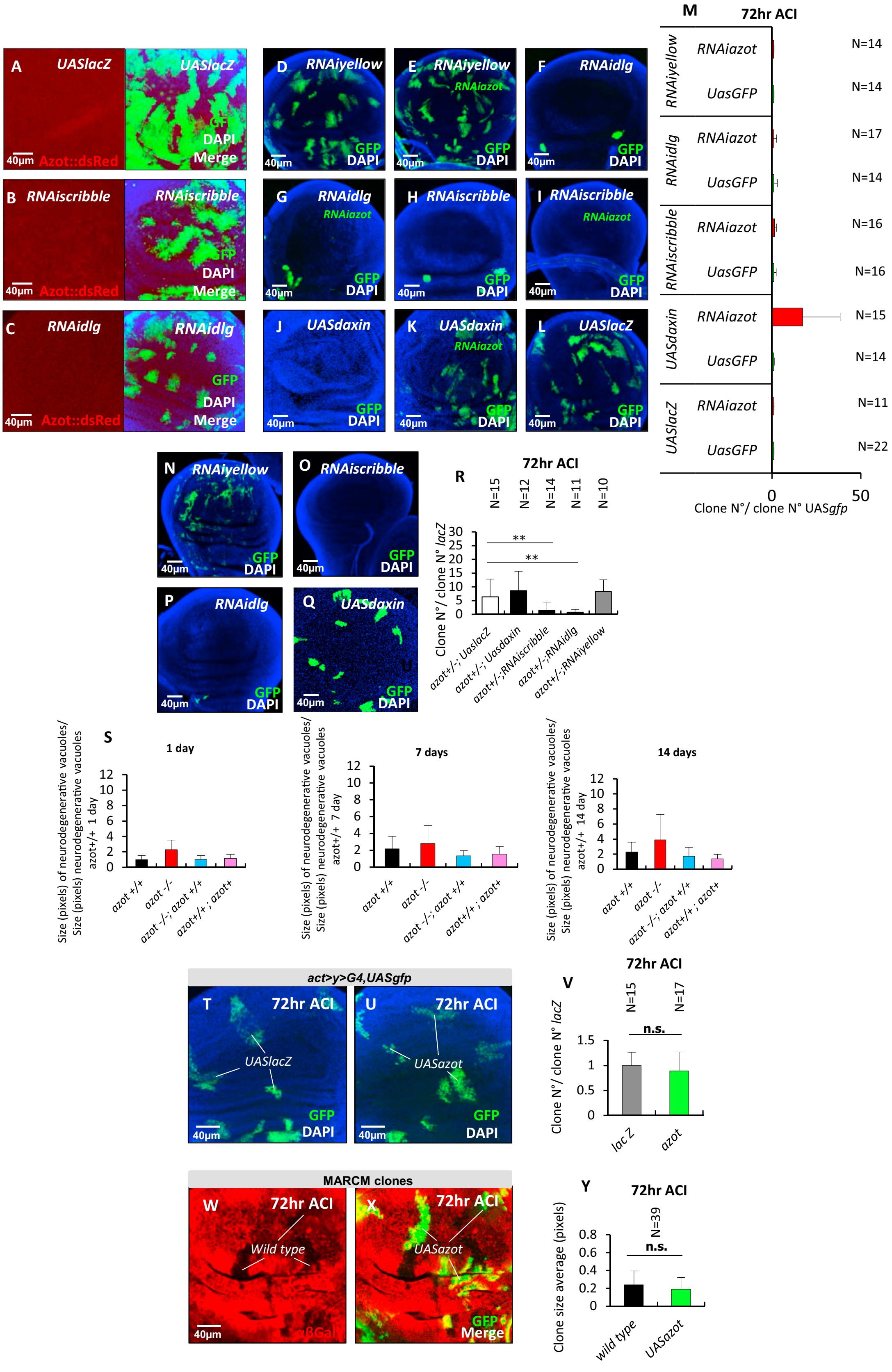

(legend on next page)

Figure S6. Expression and Functional Analysis of *azot* in Cell Clones with Defects in Apicobasal Polarity, Vacuole Size, and Clone Analysis for *azot* Overexpression, Related to Figure 6

All images are wing imaginal discs dissected in third instar larvae.

(A–C) Expression analysis of Azot (48hr ACI) of the following genotypes: hsflp; act > y+STOP > Gal4,UASgfp; UASlacZ (A), hsflp; act > y+STOP > Gal4,UASgfp; RNAiscribble (B) and hsflp; act > y+STOP > Gal4,UASgfp; RNAidlg (C). Azot expression is shown in red, GFP clones in green.

(D–M) RNAi-mediated silencing of azot in clones with defects in apico-basal polarity and clones deficient for Wg signaling. Quantification and images of GFP marked clones from the following genotypes: (D and M) hsflp; act > y+STOP > Gal4; RNAiyellow (E and M) hsflp; act > y+STOP > Gal4; RNAi azot; RNAiyellow, (F and M) hsflp; act > y+STOP > Gal4; RNAidlg, (G and M) hsflp; act > y+STOP > Gal4; RNAiazot; RNAidlg, (H and M) hsflp; act > y+STOP > Gal4; RNAiscribble, (I and M) hsflp; act > y+STOP > Gal4; RNAiazot; RNAiscribble, (J and M) hsflp; act > y+STOP > Gal4; UASdaxin, (K and M) hsflp; act > y+STOP > Gal4;RNAiazot; UASdaxin ($p < 0.01$) and (L and M) hsflp; act > y+STOP > Gal4; UASlacZ. All clones analyzed 72hr ACI.

(N–R) Survival analysis of clones with defects in apico-basal polarity and clones deficient for Wg signaling in azot mutant heterozygote background. Number of GFP marked clones 72hr ACI of the following genotypes: (N,R) hsflp; act > y+STOP > Gal4,UASgfp,azot^{-}; RNAiyellow, (O,R) hsflp; act > y+STOP > Gal4,UASgfp,azot^{-}; RNAiscribble ($p < 0.01$), (P,R) hsflp; act > y+STOP > Gal4,UASgfp,azot^{-}; RNAidlg ($p < 0.01$) and (Q and R) hsflp; act > y+STOP > Gal4,UASgfp,azot^{-}; UASdaxin.

(S) Vacuole size over time of the following 4 genotypes: 1) azot$^{+/+}$, 2) azot$^{-/-}$, 3) azot$^{-/-}$; azot$^{+/+}$, and 4) azot$^{+/+}$; azot^{+}. Size of degenerative vacuoles (pixels) after 1 day at 29°C (azot$^{+/+}$ n = 29, azot$^{-/-}$ N = 31, azot$^{-/-}$;azot$^{+/+}$ N = 23 and azot$^{+/+}$; azot^{+} N = 21). Size (pixels) of degenerative vacuoles per brain area after 7 days at 29°C (azot$^{+/+}$ N = 32, azot$^{-/-}$ N = 23, azot$^{-/-}$;azot$^{+/+}$ N = 16 and azot$^{+/+}$; azot^{+} N = 39). Size (pixels) of degenerative vacuoles per brain area after 14 days at 29°C (azot$^{+/+}$ N = 34, azot$^{-/-}$ N = 34, azot$^{-/-}$;azot$^{+/+}$ N = 22 and azot$^{+/+}$; azot^{+} N = 31).

(T–V) Images of wing imaginal discs dissected in third instar larvae and quantification of GFP marked clones. (T, U) Wing discs of the following genotypes: hsflp; act > y+STOP > Gal4, UASgfp;UASlacZ (T) and hsflp; act > y+STOP > Gal4, UASgfp;UASazot (U) 72hr ACI. Clones shown in green (GPF) and nuclear marker DAPI in blue. (V) Graph showing the quantification of the number of clones 72hr ACI. No significant differences were found (student's t test, $p > 0.05$).

(W–Y) Images of wing imaginal discs dissected in third instar larvae and quantification of clone size using the MARCM technique. (W and X) Wing discs 72hr ACI. Size of the clones shown in green (GPF) overexpressing UASazot were compared to MARCM twin clones (black), anti-βGal (red, W) and merge with GFP (X). (Y) Graph showing the quantification of the size of clones 72hr ACI. No significant differences were found (student's t test, $p > 0.05$).

Data are represented as mean ± SEM.

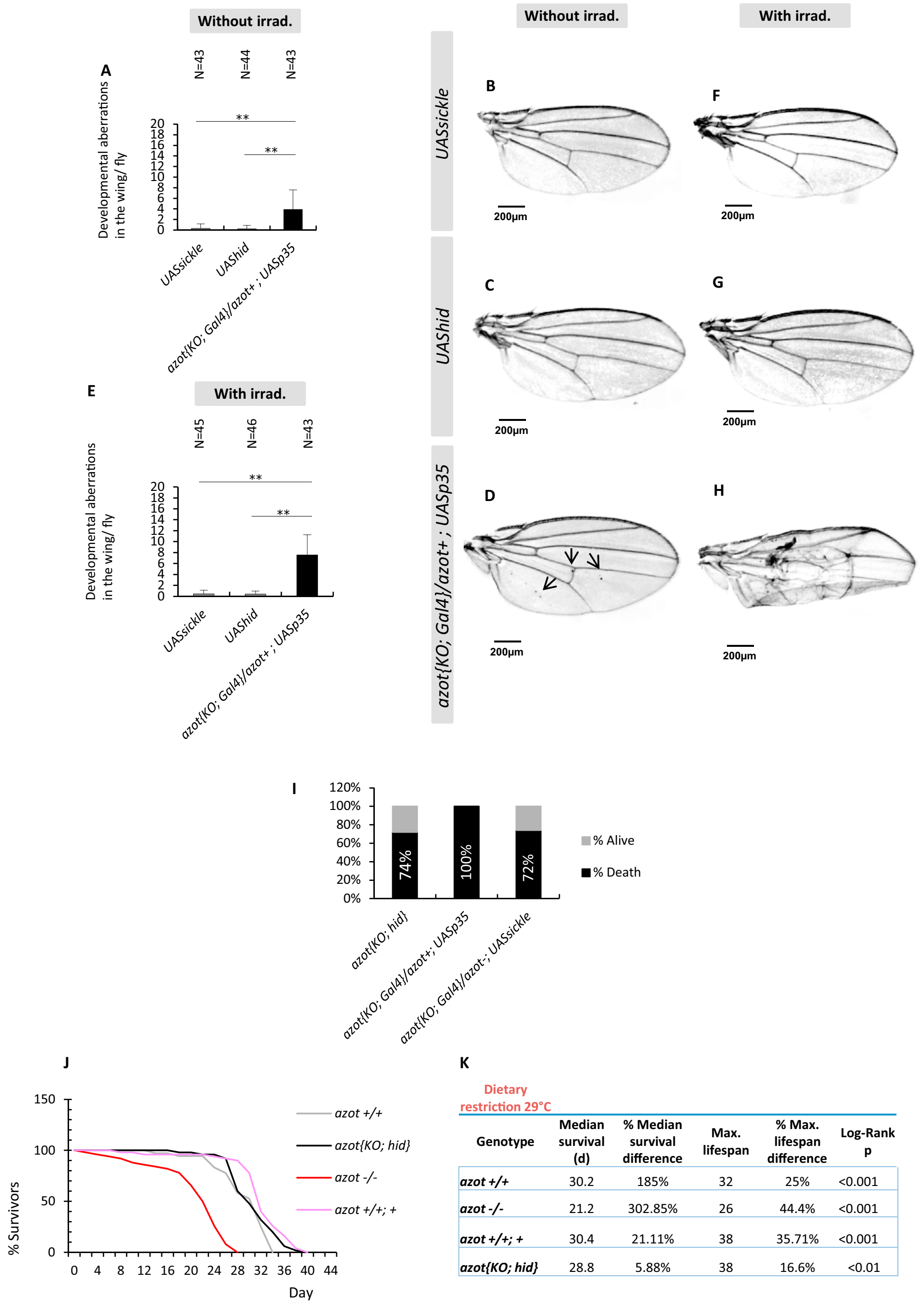

Genotype	Median survival (d)	% Median survival difference	Max. lifespan	% Max. lifespan difference	Log-Rank p
azot +/+	30.2	185%	32	25%	<0.001
azot -/-	21.2	302.85%	26	44.4%	<0.001
azot +/+; +	30.4	21.11%	38	35.71%	<0.001
azot{KO; hid}	28.8	5.88%	38	16.6%	<0.01

Figure S7. Developmental Aberrations when Inhibiting Apoptosis of Azot-Positive Cells, Related to Figure 7

(A–D) Quantification and *Drosophila* wing images of developmental aberrations, before irradiation treatment of the following genotypes: (B) *UASsickle,* (C) *UAShid* and (D) *azot{KO; Gal4}/azot*$^{+}$ *;UASp35*.

(E–H) Quantification and *Drosophila* wing images of developmental aberrations, after UV-irradiation of the following genotypes: (F) *UASsickle,* (G) *UAShid* and (H) *azot{KO; Gal4}/azot*$^{+}$*;UASp35*. Irradiation dose of 2x10^{-2}J administered during pupal stage 0. All wings belong to 10-13 days old flies.

(I) Percentage of adult survival 6 days post-irradiation (5x10^{-2}J, 1-3 days old) of the following 3 genotypes: 1) *azot{KO;hid}/azot{KO;hid},* 2) *azot{KO; Gal4}/azot*$^{+}$*; UASp35* and 3) *azot{KO; Gal4}/azot*$^{-}$*; UASsickle*.

(J and K) Dietary restriction lifespan studies at 29°C of the following 4 genotypes over time: 1) *azot*$^{+/+}$, 2) *azot*$^{-/-}$, 3) *azot*$^{+/+}$*; azot*$^{+}$ and 4) *azot{KO;hid}/ azot {KO;hid}*. (K) Median and maximum survival of the four genotypes.

Data are represented as mean ± SEM.
